# *DUOX2* and *DUOXA2* Variants Confer Susceptibility to Thyroid Dysgenesis and Gland-*in-situ* With Congenital Hypothyroidism

**DOI:** 10.3389/fendo.2020.00237

**Published:** 2020-04-21

**Authors:** Fengqi Wang, Yucui Zang, Miaomiao Li, Wenmiao Liu, Yangang Wang, Xiaolong Yu, Hua Li, Fang Wang, Shiguo Liu

**Affiliations:** ^1^Medical Genetic Department, The Affiliated Hospital of Qingdao University, Qingdao, China; ^2^Prenatal Diagnosis Center, The Affiliated Hospital of Qingdao University, Qingdao, China; ^3^Department of Endocrinology, The Affiliated Hospital of Qingdao University, Qingdao, China; ^4^Department of Rheumatology and Immunology, The Affiliated Hospital of Qingdao University, Qingdao, China

**Keywords:** congenital hypothyroidism, thyroid dysgenesis, gland-*in-situ*, multiple allele, *DUOX2*, *DUOXA2*

## Abstract

**Background:** Thyroid dysgenesis (TD), which is caused by gland developmental abnormalities, is the most common cause of congenital hypothyroidism (CH). In addition, advances in diagnostic techniques have facilitated the identification of mild CH patients with a gland-*in-situ* (GIS) with normal thyroid morphology. Therefore, TD and GIS account for the vast majority of CH cases.

**Methods:** Sixteen known genes to be related to CH were sequenced and screened for variations by next-generation sequencing (NGS) in a cohort of 377 CH cases, including 288 TD cases and 89 GIS cases.

**Results:** In our CH cohort, we found that *DUOX2* (21.22%) was the most commonly variant pathogenic gene, while *DUOXA2* was prominent in TD (18.75%) and *DUOX2* was prominent in GIS (34.83%). Both biallelic and triple variants of *DUOX2* were found to be most common in children with TD and children with GIS. The most frequent combination was *DUOX2* with *DUOXA1* among the 61 patients who carried digenic variants. We also found for the first time that biallelic *TG, DUOXA2*, and *DUOXA1* variants participate in the pathogenesis of TD. In addition, the variant p.Y246X in *DUOXA2* was the most common variant hotspot, with 58 novel variants identified in our study.

**Conclusion:** We meticulously described the types and characteristics of variants from sixteen known gene in children with TD and GIS in the Chinese population, suggesting that *DUOXA2* and *DUOX2* variants may confer susceptibility to TD and GIS via polygenic inheritance and multiple factors, which further expands the genotype-phenotype spectrum of CH in China.

## Introduction

Congenital hypothyroidism (CH), a common childhood disease associated with mental disabilities, manifests as high thyroid-stimulating hormone (TSH) levels and low T4 levels in the serum during neonatal screening due to insufficient production of thyroid hormone. CH can lead to short stature and intellectual disability if not diagnosed and treated at an early stage (1–4). Based on the Chinese neonatal screening program that started in 1984, CH affected up to 1/2421 neborns (4.13 per 10,000 live births) between 2013 and 2015, compared to 1/3000–1/4000 newborns worldwide; these figures suggest a high incidence in China and prompted us to investigate the pathogenesis of this disease (5–7).

Thyroid dysgenesis (TD), the most common cause of CH, is caused by the failure of the thyroid gland to develop a normal morphology or position and encompasses the spectrum of thyroid agenesis, thyroid ectopy, and thyroid hypoplasia (8, 9). Insufficient hormone production secondary to TD is mainly due to defects in genes that encode enzymes that participate in iodine organification, thyroglobulin synthesis or transport, iodine transport or iodotyrosine deiodination during thyroid hormone synthesis (10). Although gland-*in-situ* (GIS) with normal thyroid morphology and goiter were once thought to be present in only 20–30% of CH patients, an increase in the frequency of CH with GIS has been reported, with the incidence more than doubled to ~1 in 1,500 live newborns, which accounted for almost two-thirds of diagnosed cases in Italy at the time (11, 12). Increasing evidence has shown that the pathogenesis of CH is complex, and this disease is considered to be influenced by environmental factors and genetic causes; genetic factors play an important role in the pathogenesis of CH (13). Therefore, studies of large cohorts and multiracial groups are required to investigate genetic causality due to the unclear underlying cause and complex etiology of CH.

Thyroid morphogenesis is a multistage process that proceeds from thyroid progenitors to the thyroid gland, and thyroid-stimulating hormone receptor *(TSHR)*, thyroid transcription factors, such as *FOXE1, NKX2-1 PAX8*, and *NKX2-5*, and transcription regulators, such as *GLIS3*, are required for the normal development of the thyroid gland; mutations in these factors can lead to the failure of normal thyroid formation (14–16). Dyshormonogenesis is often due to genetic defects in important enzymes in thyroid hormone synthesis: iodine organification disorder (*TPO, DUOX2, DUOXA2*, and *SLC26A4*); thyroglobulin synthesis or transport defect (*TG*); iodine transport defect (*SLC5A5*); and iodotyrosine deiodinase deficiency (*IYD*) (17–20). In addition, a very small proportion of CH children are severely resistant to thyroid hormones due to variants in *THRB* (21). Although genetic variants of candidate genes are involved in thyroid hormone synthesis and *TSHR* and intersect with genes associated with both TD and dyshormonogenesis (19, 22), the genetic basis of GIS remains poorly understood.

Investigations of the genetics of TD and dyshormonogenesis have revealed that the genetic diversity of relevant genes far exceeds our expectations. Previous studies have applied next-generation sequencing (NGS) as a cost-efficient and powerful approach and confirmed that the conventional goiter-associated candidate genes *DUOX2* and *TPO* may be related to TD, suggesting that the genotype-phenotype relationship of CH is increasingly complex (23–26). However, most previous studies focused on only a single gene or a small number of genes and thus had significant limitations in elucidating the relationship between genotype and phenotype. Therefore, it is necessary to explore the comprehensive effects and variant rates of these causative genes in sporadic cases as part of an in-depth study of the pathogenesis of CH. In this study, we will explore 16 causative genes associated with CH by conducting NGS in 377 CH cases comprising 288 TD cases and 89 GIS cases in China to reveal the genetic complexity of CH, which will expand the genetic spectrum of this disease and clarify the interactions among multiple genes.

## Materials and Methods

### Editorial Policies and Ethical Considerations

Our study was approved by the ethics committee of the Affiliated Hospital of Qingdao University. Written informed consent was provided by the parents of the participants.

### Study Samples

A total of 377 children with sporadic CH, including 288 children with TD (121 athyreosis, 93 ectopia, and 74 hypoplasia) and 89 children with GIS were recruited from newborn screening centers in 11 cities in Shandong, China (Qingdao, Zibo, Jining, Linyi, Yantai, Jinan, Liaocheng, Dezhou, Weifang, Zaozhuang and Tai'an) (Table S1). The clinical, biochemical and ultrasonographic criteria of the patients were as follows: (1) All patients were full-term children aged 1–6 years old and had no other congenital diseases. (2) Heel blood was collected within 72 h after birth and TSH levels were analyzed. The children with TSH > 10 μL/ml were recalled within 2 months after birth. The levels of serum TSH, FT4, and FT3 were further measured. Then, CH was diagnosed according to high TSH (> 4.2 μIU/ml) and low FT4 (<12 μpmol/L). (3) The morphological classification of thyroid is as follows (shown by ultrasonography or technetium-99 m thyroid scanning): (a) GIS means a child diagnosed with CH, accompanied by normal thyroid anatomy and imaging findings; (b) athyreosis is defined as failure to show any thyroid tissue on ultrasonography and technetium thyroid scans; (c) ectopia refers to the absence of thyroid tissue in normal thyroid anatomical structure, but appeared in other areas; (d) hypoplasia refers to unilateral and/or bilateral thyroid gland not reaching normal size and lacking normal shape (27). Thyroid morphology was classified by thyroid ultrasonography and technetium thyroid scans (28, 29). In addition, we excluded children with chromosomal abnormalities, children with central hypothyroidism or associated syndromes and offspring of mothers with Graves' disease. All subjects represented sporadic and non-consanguineous cases.

### Targeted NGS

A Qiagen DNA extraction kit (Qiagen, Hilden, Germany) was used to extract DNA samples from blood clots using the column method. The following is the process of library preparation: (1) genomic DNA randomly fragmented to an average size of 180–280 bp; (2) gDNA fragments repaired and phosphorylated; (3) A-tailing and ligation at the 3′ends with paired-end adaptors(Illumina) with a singel “T” base overhang and purification using AMPure SPRI beads from Agencourt. DNA library was hybridized with the probe library labeled with biotin in liquid phase. The probe library was specifically combined with the exons of 16 candidate genes: *PAX8* (NM_013952), *NKX2-5* (NM_004387), *GLIS3* (NM_001042413), *FOXE1* (NM_004473), *NKX2-1* (NM_003317), *TSHR* (NM_000369), *THRB* (NM_000461), *DUOX2* (NM_014080), *DUOXA2* (NM_207581), *DUOXA1* (NM_001276265), *DUOX1* (NM_175940), *TG* (NM_003235), *TPO* (NM_001206745), *SLC5A5* (NM_000453), *IYD* (NM_001164695; NM_203395), and *SLC26A4* (NM_000441). Then, the prepared library was sequenced on an Illumina Hiseq x for 150 bp paired-end reads.

### Data Filtering and Variant Calling

The raw image files obtained from the Hiseq 4,000 were processed with an Illumina pipeline for base calling and stored in FastQ format (raw data). Adapter contamination, uncertain nucleotides >10% and single read >50% low quality were used to filter the data. The filtered data were mapped to the reference genome (UCSC hg19) by Burrows-Wheeler Aligner (BWA) software (version 0.7.8–r455) to obtain the original mapping result in the Binary Alignment/Map (BAM) format. SAMtools (0.1) and Picard (1.111) were utilized to sort BAM files and mark duplicate reads, respectively. Reads that aligned to exon regions were collected by SAMtools mpileup and BCFtools for variant calling and single-nucleotide polymorphism (SNP) and indel identification. After SAMtool calling, the variants were annotated by ANNOVAR (ANNOtate VARiation) software (30).

### Variant Annotation

The variant position, variant type, conservative prediction and other information were annotated by ANNOVAR through a variety of databases, such as DbSNP, 1,000 Genome, ExAC, CADD and HGMD. We defined the variations with the following criteria: (1) quality scores >30, site read depth >10×, variant depth >5×, and mapping quality >50; (2) minor allele frequency (MAF) < 0.01 in the 1,000 Genomes databases and ExAC; (3) at least half of the employed algorithms (PolyPhen-2, SIFT, Mutation Taster and CADD) showed that the single-nucleotide variant (SNV) results in an amino acid change or is not benign (defined as a nonsense or splice site variant). All of these variants were verified by Sanger sequencing.

## Results

### NGS Identified Likely Pathogenic Variations in Sixteen Genes in CH-Affected Subjects

We detected variants in 16 konwn genes and identified 168 likely pathogenic variations, including 12 splicing variants, 18 nonsense variants and 138 missense SNVs, which were further validated by Sanger sequencing of 189 variants in 219 of 377 patients (58.09%). Patients carrying variants were as follows: 62 of 89 GIS patients (69.66%) and 157 of 288 TD patients (54.51%), with 65 of 121 athyreosis patients (53.72%), 48 of 93 ectopia patients (51.61%), and 44 of 74 hypoplasia patients (59.46%) (Table S1, Table 1).

**Table 1:** Variant rates of candidate genes and biallelic or multiallelic variants in the cohort.

**Gene**	**Number of variants**	**Variant type**	**Number in All**	**Number in TD**	**Number in TD**			**Number in Gland-*in-situ* (GIS)**	**Biallelic mutation in TD**	**Biallelic mutation in GIS**	**Triallelic mutation in TD**	**Triallelic mutation in GIS**
					**Athyreosis**	**Ectopia**	**Hypoplasia**					
PAX8	4	3nonsense + 1missense SNV	5/377	4/288	1/121	1/93	2/74	1/89				
THRB	4	1splicing + 3missense SNV	5/377	5/288	–	2/93	3/74	–				
NKX2-5	2	2missense SNV	2/377	2/288	1/121	–	1/74	–				
GLIS3	10	9missense SNV + 1nonsense	12/377	10/288	6/121	2/93	2/74	2/89				
FOXE1	6	6missense SNV	8/377	7/288	1/121	5/93	1/74	1/89				
NKX2-1	4	4missense SNV	12/377	8/288	5/121	1/93	2/74	4/89				
TSHR	21	2splicing + 1nonsense + 18missense SNV	33/377	24/288	8/121	6/93	10/74	9/89	6/288	4/89		
DUOX2	45	3splicing + 3nonsense + 39missense SNV	80/377	49/288	20/121	14/93	15/74	31/89	6/288	9/89	3/288	3/89
DUOXA2	7	2splicing + 2nonsense + 3missense SNV	75/377	54/288	22/121	17/93	15/74	21/89	1/288	1/89		
DUOXA1	3	3missense SNV	34/377	21/288	5/121	9/93	7/74	13/89	1/288			
DUOX1	7	7missense SNV	8/377	5/288	2/121	2/93	1/74	3/89				
TG	29	2splicing + 7nonsense + 20missense SNV	44/377	32/288	17/121	7/93	8/74	12/89	2/288			
TPO	11	1splicing + 10missense SNV	15/377	10/288	2/121	3/93	5/74	5/89		1/89		
SLC5A5	3	1nonsense + 2missense SNV	3/377	3/288	2/121	1/93	–	–				
IYD	3	3missense SNV	4/377	3/288	3/121	–	–	1/89				
SLC26A4	9	1splicing + 8missense SNV	13/377	11/288	5/121	5/93	1/74	2/89				
Total	168	12splicing + 18nonsense + 138missense SNV	219/377	157/288	65/121	48/93	44/74	62/89	16/288	15/89	3/288	3/89

We found that the most commonly gene with variants was *DUOX2* (21.22%, 80/377), which contained 45 variants comprising 3 splicing variants, 3 nonsense variants and 39 missense SNVs. Forty-nine of 288 children with TD carried *DUOX2* variants, including 20 of 121 children with athyreosis, 14 of 93 children with ectopia and 15 of 74 children with hypoplasia, and 31 of 89 children with GIS carried *DUOX2* variants. After *DUOX2, DUOXA2* was the second most commonly gene with variants (19.89%, 75/377), including 2 splicing variants, 2 nonsense variants and 3 missense SNVs. Among 288 children with TD, 22 of 121 children with athyreosis, 17 of 93 children with ectopia and 15 of 74 children with hypoplasia carried *DUOXA2* variants. Among the 89 children with GIS, 21 children with *DUOXA2* variants were identified. *DUOXA2* was followed by *TG* (11.67%, 44/377), *DUOXA1* (9.02%, 4/377), and *TSHR* (8.75%, 33/377) (Table 1).

We also found that *DUOXA2* variants (18.75%, 54/288) were prominent in patients with TD, followed by *DUOX2* (17.01%, 49/288); *DUOX2* variants (34.83%, 31/89) were followed by *DUOXA2* variants (23.60%, 21/89) in patients with GIS (Table 1). We performed a Pearson chi-square analysis of *DUOX2* variant rates and found significant differences between TD patients and GIS patients (Pearsonχ^2^ = 12.911, *p*-value= 3.27 × 10^−4^).

### Distribution of Biallelic or Multiallelic Variants in the Cohort

**Figure 1:**
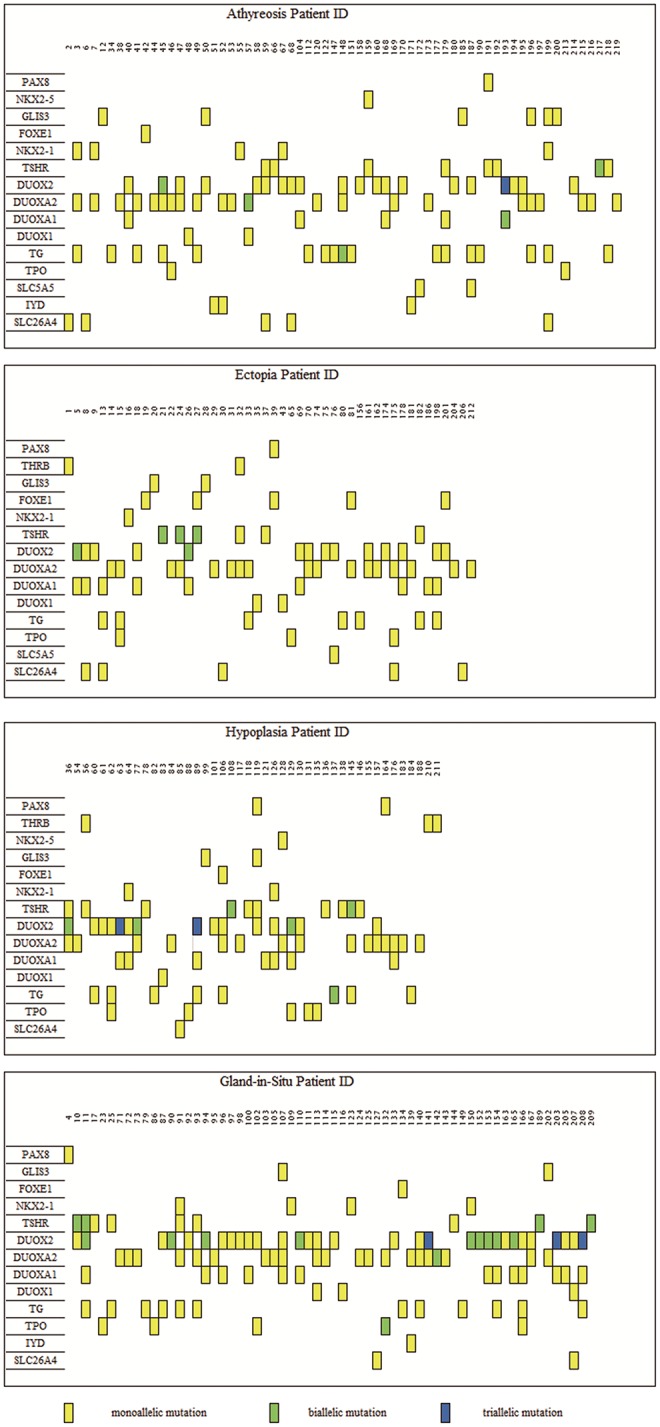
Distribution of variants in 219 congenital hypothyroidism. The left side was 16 detected genes with variants, and the number on top of each box is the patient ID. Each column represents a patient and each row represents a gene. Blue blocks represent triallelic variants, green blocks represent biallelic variants and yellow blocks represent monoallelic variants. Such as patient 45 carries variants on three genes: biallelic variants in the DUOX2 gene and each monoallelic variant in TG and *DUOXA2*. A total of 80 subjects bore *DUOX2* variants, which was the most commonly pathogenic genes with variants in our study. As for TD (encompassing thyroid, ectopy and hypoplasia), the most frequently was *DUOXA2*, which held 54 carriers.

To characterize the variant rates of biallelic and multiallelic variants (2 or more pathogenic variants on the same allele) of these sixteen genes in the cohort, we identified biallelic variants in 16 of 288 children with TD (5.56%) and 15 of 89 children with GIS (16.85%) (Pearsonχ^2^ = 11.501, *p*-value=6.96 × 10^−4^). These variants were distributed in the following genes: *DUOX2* (2.08%, 6/288), *TSHR* (2.08%, 6/288), *TG* (0.69%, 2/288), *DUOXA2* (0.35%, 1/288), and *DUOXA1* (0.35%, 1/288) in patients with TD and *DUOX2* (10.11%, 9/89), *TSHR* (4.49%, 4/89), *DUOXA2* (1.12%, 1/89), and *TPO* (1.12%, 1/89) in patients with GIS. Furthermore, 3 cases of triple variants (3 variants on the same allele) in *DUOX2* were found in children with TD (1.04%, 3/288) and children with GIS (3.37%, 3/89) (Table 1, Figure 1).

### Variant of Multiple Genes Was Detected in our 94 Subjects

**Figure 2:**
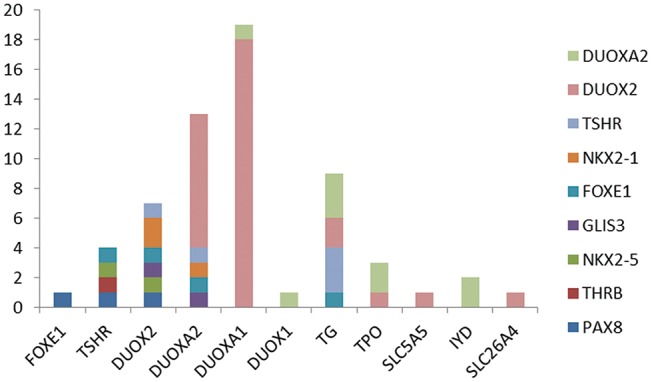
The 61 digenic variants in our CH cohort. Each color lump of a column represents a combination of a biallelc variant. For example, there are four genes with variants at the same time with *TSHR*, which are one in each of *FOXE1, PAX8, THRB, NKX2-5*, and two case is the combination of *IYD* and *NKX2-5*. From the picture, we can see that the most frequently combination was *DUOX2* and *DUOXA1*.

Because more than one gene can participate in the process of CH in an individual and because CH is a complex disease involving multiple genes (31, 32), we analyzed the sequencing information for subjects carrying two or more genes with variants and conducted an analysis of various gene combinations (Table 2). Among the 61 digenic variants, the most frequent combination was *DUOX2* with *DUOXA1* (29.51%, 18/61 in all patients; 8/19 in GIS patients and 10/42 in TD patients) (Figure 2); among 26 trigenic variants, the most common combination was *DUOX2* and *TG* combined with *DUOXA1* (15.38%, 4/26) or *DUOXA2* (11.54%, 3/26), and 7 subjects had joint variants of four genes (Table S1, Patient ID: 11; 91; 106; 107; 119; 166; and 199).

**Table 2:** Multiple genes with variants simultaneous in our subjects.

**Number of gene with variants**	**Athyreosis**	**Ectopia**	**Hypoplasia**	**Gland *in situ* (GIS)**	**Number in TD**	**Number in GIS**
1	39	27	26	33	92/288	33/89
2	18	15	9	19	42/288	19/89
3	7	6	7	6	20/288	6/89
4	1	0	2	4	3/288	4/89
Total	65	48	44	62	157/288	62/89

### We Detected 58 Novel Variants and Found 10 Possible Variant Hotspots in our CH Cohort

Among the 168 variants, 58 novel variants were identified, as follows: 12 variants in *TG*; 11 variants in *DUOX2*; 8 variants in *TSHR*; 5 variants in *DUOXA1*; 3 variants each in *PAX8, SLC5A5, DUOXA2* and *TPO*; 2 variants each in *GLIS3, NKX2-1* and *THRB*; and 1 variant each in *FOXE1, IYD, NKX2-5*, and *SLC26A4* (Table S2). We found 10 possible variant hotspots of these sixteen genes that were concentrated significantly in the Chinese CH population (Table 3). Specifically, a nonsense (p.Y246X) variant in *DUOXA2* had an extremely high rate, with a frequency of up to 68 in 377 cases (18.04%), including 20 GIS cases and 48 TD cases (comprising 13 hypoplasia cases, 17 ectopia cases and 18 athyreosis cases). The following missense SNVs were detected: p.S268G in *DUOXA1* (4.77%, 18/377); p.T168M in *DUOXA1* (4.24%, 16/377) and 7 additional sites in *DUOX2, NKX2-1*, and *TG* (Table 3).

**Table 3:** Ten possible hotspots in our CH cohort.

**No**.	**Chromosome**	**Position**	**Gene**	**Variant**	**Variant type**	**Number of people**	**People in TD**	**People in gland-*in-situ***
1	14	36,986,635	NKX2-1	c.G964A: p.G322S	Missense SNV	8	4/288	4/89
2	14	81,609,751	TSHR	c.G1349A: p.R450H	Missense SNV	10	8/288	2/89
3	15	45,388,079	DUOX2	c.C4027T: p.L1343F	Missense SNV	14	9/288	5/89
4	15	45,391,946	DUOX2	c.G3329A: p.R1110Q	Missense SNV	14	6/288	8/89
5	15	45,396,158	DUOX2	c.G2654T: p.R885L	Missense SNV	9	5/288	4/89
6	15	45,398,423	DUOX2	c.G2048T: p.R683L	Missense SNV	8	4/288	4/89
7	15	45,409,472	DUOXA2	c.C738G: p.Y246X	nonsense	68	48/288	20/89
8	15	45,411,399	DUOXA1	c.A802G: p.S268G	Missense SNV	18	13/288	5/89
9	15	45,412,435	DUOXA1	c.C503T: p.T168M	Missense SNV	16	8/288	8/89
10	8	134,128,945	TG	c.A7847T: p.N2616I	Missense SNV	8	8/288	0

## Discussion

In our study, we conducted NGS for 16 known genes associated with CH in a total of 377 CH-affected subjects and annotated non-synonymous variants of these known genes to predict the cause of thyroid dysfunction or abnormal morphology by the highly rigorous ACMG criteria. We found that *DUOX2* was the most significantly gene with variants in our CH cohort, while *DUOXA2* was significant in TD patients and *DUOX2* was prominent in GIS patients.

We found that *DUOX2* had a significantly high rate (21.22%) in our study, mainly based on CH, with 288 TD patients (16.67%) and 89 GIS patients (34.83%), which indicated that *DUOX2* is an important susceptibility gene in the Chinese CH population. The high prevalence of *DUOX2* variant in our work is consistent with previous studies in China (variant rate of *DUOX2* from 31.8 to 62.5%), suggesting that *DUOX2* is an important causative gene involved in the pathogenesis of CH (31–33). As *DUOX2* has generally been considered a gene involved in disorders of H_2_O_2_ generation (11, 34, 35), a majority of previous studies focused on dyshormonogenesis and found that *DUOX2* pathogenic variants were present in 62.5% of the cohort consisting of 154 CH patients, with 118 dyshormonogenesis patients and 36 TD patients (33); Sun et al. (32) revealed that *DUOX2* was present in 60% of 110 CH patients, including 21 goiter patients, 51 patients with a normal-sized thyroid and 28 patients with unknown thyroid morphology. Recently, One study has shown *DUOX2* mutations associated with CH with ectopic thyroid (25). However, mouse models with biallelic inactivation of *DUOX2* or *DUOXA2* present a normal developed thyroid gland with only thyroid dyshormonogenesis (36, 37). Our study also found that the high rate of *DUOX2* in GIS patients was significantly different from that in TD (Pearsonχ^2^ = 12.911, *p*-value= 3.27 × 10^−4^), indicating that *DUOX2* is an important factor associated with GIS in China; this result differed significantly from previous research in western countries that suggested that variants of *TG* are the most common cause of GIS (19). Therefore, we speculated that *DUOX2* confers susceptibility not only to TD but also to GIS.

In our TD cohort, the most significant variant was *DUOXA2* (18.75%), which had a slightly higher rate than *DUOX2*. As a necessary maturation factor for functional *DUOX2, DUOXA2* is an accessory protein required for the correct processing of the DUOX2 enzyme and is generally involved in dyshormonogenesis (38). In our study, we first found that *DUOXA2* confers susceptibility to TD, which provides new insights for understanding thyroid development. In addition, we found that 23.6% of children with GIS carried *DUOXA2* variants. Thus, we speculate that *DUOXA2*, as a critical maturation factor for functional *DUOX2*, may play an important role in TD and GIS. We also found 10 subjects in our 288 TD cohort with *TPO* variants, which is consistent with the fact that *TPO* was previously believed to be involved in thyroid hormone synthesis, which may also lead to TD (26). Therefore, our findings contribute to the variant spectrum associated with multiple CH phenotypes in China and may explain the multigene interactions and new roles of *DUOX2* and *DUOXA2* in children with TD and GIS.

*DUOX2* encodes an enzyme responsible for catalyzing the production of H_2_O_2_ from the apical membrane of follicular epithelial cells, which is necessary for the iodization of thyroglobulin tyrosine residues. DUOXA2 proteins migrate to the cell surface with DUOX2 to constitute the functional active complex (39). DUOX2/DUOXA2 complex plays an important role in the transport, maturation and localization of DUOX2. Recently, studies have shown that duox and duoxa are new thyroid differentiation markers in zebrafish, and duoxa is earlier expressed than duox (40). It has also been proved in insects that the main purpose of duox activity is to guarantee tyrosine residues cross-linked, which is helpful for the cuticle formation in Drosophila (41). *DUOX2* is one downstream target of *FOXE1* in rat thyroid follicular cells (pccl3) (42). This suggests that duox family may play an additional role in thyroid development by interacting with other undetermined proteins.

We found that biallelic variants were present in 5.56% (16 of 288) of children with TD and 16.85% (15 of 89) of children with GIS in our study, and this difference was statistically significant (Pearsonχ^2^ = 11.501, *p*-value=6.96 × 10^−4^), which indicated that biallelic variants may play a key role in GIS. The most common biallelic variants involved *DUOX2* in children with GIS, and *DUOX2* or *TSHR* in children with TD. We showed for the first time that biallelic variants of *TG, DUOXA2* and *DUOXA1* participated in the pathogenesis of TD. We also identified triple variants in *DUOX2* in three cases each of TD and GIS. However, it is uncertain whether the phenotypic severity of CH depends on the number of allelic variants. Further studies in larger families are needed to assess the contributions of different variables to the phenotype of CH.

We detected monogenic variants of 16 known genes in 125 cases (26 athyreosis, 21 ectopia, 18 hypoplasia and 29 GIS) and non-monogenic variants in 94 cases (39 athyreosis, 27 ectopia, 26 hypoplasia and 33 GIS). The most frequently digenic variant was *DUOX2* combined with *DUOXA1* in both TD and GIS. However, previous studies reported that *TG* combined with *DUOX2* had the most significant variant rate in children with GIS and dyshormonogenesis (19, 43). Hulur et al. (44) reported that a CH child with goiter lacked one allele of *DUOX2* and *DUOXA1*, with loss of function of *DUOXA2*. We demonstrated for the first time that *DUOX2* and *DUOXA1*, as members of the NADPH oxidase family that were once thought to be involved only in thyroid hormone synthesis (35, 45), may play a role in thyroid development. Furthermore, we found 26 subjects carrying trigenic variants mostly in *DUOX2* and *TG* accompanied by *DUOXA1/DUOXA2*, and 7 cases had joint variants of four genes without obvious gene aggregation. These diverse genetic variant combinations indicated that CH is a complex genetic disease.

In this study, we found that there are two or more pathogenic variants on the same allele and a large number of multigenic pathogenic variants. As recently reported (46), mutations of multiple genes cause phenotype aggravation because of the weakening of the attenuated compensation effect of related genes. In fact, environmental factors, such as iodine deficiency disorders, may cause endocrine dysfunction by affecting gene expression. Another article revealed that (47) the frequent combination of rare variations in CH patients was independent of phenotype, implying CH has a oligogenic origin, which can explain the absence of CH heritability and sporadic manifestations of the disease. We support the view that CH was an oligogenetic predisposition disease with multiple defects in different loci or genes in sporadic cases.

We identified a high prevalence of p.Y246X variants in *DUOXA2* in our 68 subjects, but this hotspot is quite different from the p.K530X variants in *DUOX2* in Guangzhou due to possible differences in population composition between North and South China (33). In addition, we detected p.S268G and p.T168M in *DUOXA1* with other hotspots. The variant hotspots in these genes laid the foundation for future genetic screening.

In summary, our research made full use of the superiority of NGS to identify pathogenicity variants in 16 known genes based on CH in a Chinese population. We meticulously described the types and characteristics of gene variants in TD and GIS patients in the Chinese population, suggesting that *DUOXA2* and *DUOX2* variants may confer susceptibility to TD and GIS via polygenes and multiple factors, which further expands the genotype-phenotype spectrum of CH in China. However, genetic verification in large families and functional research on the identified variants are necessary for further insights into the etiology of CH.

## Data Availability Statement

The datasets presented in this study can be found in online repositories. The names of the repository/repositories and accession number(s) can be found below: NCBI SRA: PRJNA613190.

## Ethics Statement

The studies involving human participants were reviewed and approved by the ethics committee of the Affiliated Hospital of Qingdao University. Written informed consent was provided by the parents of the participants.

## Author Contributions

SL and FaW selected topics, and participated in design and coordination. FeW analyzed the data and drafted the manuscript. YZ, ML, and WL collected the sample and analyzed the basic clinical data. YW, HL, and XY interpreted the results and provided critical revisions of the manuscript. All authors approved the final version of the manuscript and agreed to be accountable for all aspects of the work.

## Conflict of Interest

The authors declare that the research was conducted in the absence of any commercial or financial relationships that could be construed as a potential conflict of interest.
