# Supplementary material for: DUOX2 and DUOXA2 Variants Confer Susceptibility to Thyroid Dysgenesis and Gland-in-situ With Congenital Hypothyroidism
Source: Front Endocrinol (Lausanne). 2020 Apr 21;11:237. doi: 10.3389/fendo.2020.00237 (PMC7212429; doi:10.3389/fendo.2020.00237)
Supplement: Supplementary file 1 [file Table_1.DOCX]

Table S1: Clinical and mutation information of 219 mutated CH patients in our cohort

| Patient ID | Gender | Thyroid morphology | Chromosome | Position | Gene | Varient | Varient Type | AF in 1000G | AF in ExAC | CADD | SIFT | Polyphen2 | Mutation Taster | GERP++ |
| --- | --- | --- | --- | --- | --- | --- | --- | --- | --- | --- | --- | --- | --- | --- |
| 1 | female | Ectopia | 3 | 24231741 | *THRB* | c.G107A:p.C36Y | missense SNV | NA | 0.0007 | 15.84 | D | B | N | 5.05 |
| 2 | male | Athyreosis | 7 | 107342497 | *SLC26A4* | c.C2029T:p.R677W | missense SNV | NA | 0 | 23.8 | D | D | N | NA |
| 3 | female | Athyreosis | 14 | 36986635 | *NKX2-1* | c.G964A:p.G322S | missense SNV | 0.003 | 0.0039 | 13.89 | T | B | D | 2.65 |
|  |  |  | 15 | 45409472 | *DUOXA2* | c.C738G:p.Y246X | nonsense | NA | 0.0025 | 34 | NA | NA | D | NA |
|  |  |  | 8 | 133900333 | *TG* | c.C2281T:p.P761S | missense SNV | 0.001 | 0.0001 | 23.3 | D | D | D | 5.81 |
| 4 | female | Gland-in-Situ | 2 | 113994290 | *PAX8* | c.C786A:p.Y262X | nonsense | NA | NA | 38 | NA | NA | A | 2.07 |
| 5 | male | Ectopia | 15 | 45388079 | *DUOX2* | c.C4027T:p.L1343F | missense SNV | 0.005 | 0.0068 | 29.5 | T | P | D | 5.57 |
|  |  |  | 15 | 45404082 | *DUOX2* | c.A397T:p.I133F | missense SNV | NA | NA | 29.1 | D | D | D | 5.22 |
|  |  |  | 15 | 45411399 | *DUOXA1* | c.A802G:p.S268G | missense SNV | 0.0088 | 0.0032 | 23.4 | D | D | D | 5.31 |
| 6 | female | Athyreosis | 7 | 107342443 | *SLC26A4* | c.G1975C:p.V659L | missense SNV | 0.001 | 0.0001 | 25.6 | D | P | D | 4.63 |
| 7 | male | Athyreosis | 14 | 36986635 | *NKX2-1* | c.G964A:p.G322S | missense SNV | 0.003 | 0.0039 | 13.89 | T | B | D | 2.65 |
|  |  |  | 15 | 45409472 | *DUOXA2* | c.C738G:p.Y246X | nonsense | NA | 0.0025 | 34 | NA | NA | D | NA |
| 8 | male | Ectopia | 15 | 45388079 | *DUOX2* | c.C4027T:p.L1343F | missense SNV | 0.005 | 0.0068 | 29.5 | T | P | D | 5.57 |
|  |  |  | 15 | 45411399 | *DUOXA1* | c.A802G:p.S268G | missense SNV | 0.0088 | 0.0032 | 23.4 | D | D | D | 5.31 |
|  |  |  | 7 | 107350577 | *SLC26A4* | c.A2168G:p.H723R | missense SNV | 0.002 | 0.0017 | 26.8 | D | D | A | 5.51 |
| 9 | male | Ectopia | 15 | 45387642 | *DUOX2* | c.G4232A:p.C1411Y | missense SNV | NA | 0.0007 | 31 | D | D | D | 5.8 |
| 10 | female | Gland-in-Situ | 14 | 81609571 | *TSHR* | c.G1169T:p.C390F | missense SNV | NA | 0.0001 | 26.4 | D | D | D | 5.37 |
|  |  |  | 14 | 81609958 | *TSHR* | c.G1556A:p.R519H | missense SNV | NA | 0.0001 | 33 | D | D | D | 5.46 |
|  |  |  | 15 | 45387781 | *DUOX2* | c.G4093A:p.G1365R | missense SNV | NA | NA | 34 | D | D | D | 5.84 |
| 11 | female | Gland-in-Situ | 14 | 81609750 | *TSHR* | c.C1348T:p.R450C | missense SNV | NA | NA | 34 | D | D | D | 5.74 |
|  |  |  | 14 | 81609976 | *TSHR* | c.T1574C:p.F525S | missense SNV | 0.001 | 0.0017 | 24 | T | D | D | 5.46 |
|  |  |  | 15 | 45388079 | *DUOX2* | c.C4027T:p.L1343F | missense SNV | 0.005 | 0.0068 | 29.5 | T | P | D | 5.57 |
|  |  |  | 15 | 45398423 | *DUOX2* | c.G2048T:p.R683L | missense SNV | 0.003 | 0.0038 | 34 | D | D | D | 5.31 |
|  |  |  | 15 | 45411399 | *DUOXA1* | c.A802G:p.S268G | missense SNV | 0.0088 | 0.0032 | 23.4 | D | D | D | 5.31 |
|  |  |  | 8 | 134107412 | *TG* | c.G7364A:p.R2455H | missense SNV | 0.005 | 0.0092 | 31 | D | D | D | 5.17 |
| 12 | male | Athyreosis | 9 | 4286137 | *GLIS3* | c.C289T:p.R97X | nonsense | NA | 0 | 36 | NA | NA | A | 3.85 |
| 13 | male | Ectopia | 15 | 45414422 | *DUOXA1* | c.C166T:p.R56W | missense SNV | 0.003 | 0.0012 | 32 | D | D | D | 4.47 |
|  |  |  | 8 | 133980165 | *TG* | c.G5813T:p.G1938V | missense SNV | NA | NA | 24.7 | D | D | D | 4.77 |
|  |  |  | 7 | 107314782 | *SLC26A4* | c.G589A:p.G197R | missense SNV | NA | 0.0001 | 33 | D | D | D | 5.07 |
| 14 | male | Ectopia | 15 | 45409472 | *DUOXA2* | c.C738G:p.Y246X | nonsense | NA | 0.0025 | 34 | NA | NA | D | NA |
| 15 | male | Ectopia | 15 | 45409472 | *DUOXA2* | c.C738G:p.Y246X | nonsense | NA | 0.0025 | 34 | NA | NA | D | NA |
|  |  |  | 8 | 134128945 | *TG* | c.A7847T:p.N2616I | missense SNV | 0.0024 | 0.0016 | 12.35 | D | B | P | NA |
|  |  |  | 2 | 1520672 | *TPO* | c.C2365T:p.R789W | missense SNV | 0.002 | 0.0017 | 25.2 | D | D | N | 2.1 |
| 16 | male | Ectopia | 14 | 36988426 | *NKX2-1* | c.C137T:p.A46V | missense SNV | NA | NA | 13.4 | T | B | D | 4.01 |
| 17 | male | Gland-in-Situ | 14 | 81610674 | *TSHR* | c.G2272A:p.E758K | missense SNV | NA | 0.0005 | 17.32 | D | B | N | 3.19 |
| 18 | female | Ectopia | 15 | 45391946 | *DUOX2* | c.G3329A:p.R1110Q | missense SNV | 0.002 | 0.0025 | 35 | D | D | D | 5.6 |
|  |  |  | 15 | 45412435 | *DUOXA1* | c.C503T:p.T168M | missense SNV | 0.002 | 0.0036 | 29.6 | D | D | D | 5.12 |
| 19 | male | Ectopia | 9 | 100616878 | *FOXE1* | c.C682G:p.P228A | missense SNV | 0.001 | NA | 22.9 | D | B | N | 2.74 |
| 20 | male | Ectopia | 9 | 3829462 | *GLIS3* | c.C2504T:p.P835L | missense SNV | NA | 0.001 | 29.5 | D | D | D | 5.93 |
| 21 | female | Ectopia | 14 | 81609958 | *TSHR* | c.G1556A:p.R519H | missense SNV | NA | 0.0001 | 33 | D | D | D | 5.46 |
|  |  |  | 14 | 81609984 | *TSHR* | c.C1582A:p.R528S | missense SNV | NA | NA | 25.3 | D | B | D | 5.46 |
| 22 | male | Ectopia | 15 | 45409472 | *DUOXA2* | c.C738G:p.Y246X | nonsense | NA | 0.0025 | 34 | NA | NA | D | NA |
| 23 | female | Gland-in-Situ | 2 | 1500456 | *TPO* | c.C2134T:p.R712W | missense SNV | 0.005 | 0.0032 | 18.22 | T | D | N | 3.08 |
| 24 | female | Ectopia | 14 | 81558898 | *TSHR* | c.T491A:p.M164K | missense SNV | NA | NA | 27.9 | D | D | D | 5.28 |
|  |  |  | 14 | 81609751 | *TSHR* | c.G1349A:p.R450H | missense SNV | 0.001 | 0.0034 | 32 | D | D | D | 5.74 |
|  |  |  | 15 | 45409472 | *DUOXA2* | c.C738G:p.Y246X | nonsense | NA | 0.0025 | 34 | NA | NA | D | NA |
| 25 | male | Gland-in-Situ | 14 | 81609984 | *TSHR* | c.C1582A:p.R528S | missense SNV | NA | NA | 25.3 | D | B | D | 5.46 |
|  |  |  | 8 | 133898968 | *TG* | c.C1351T:p.R451X | nonsense | NA | 0.0001 | 35 | NA | NA | A | NA |
| 26 | male | Ectopia | 15 | 45388079 | *DUOX2* | c.C4027T:p.L1343F | missense SNV | 0.005 | 0.0068 | 29.5 | T | P | D | 5.57 |
|  |  |  | 15 | 45404082 | *DUOX2* | c.A397T:p.I133F | missense SNV | NA | NA | 29.1 | D | D | D | 5.22 |
|  |  |  | 15 | 45411399 | *DUOXA1* | c.A802G:p.S268G | missense SNV | 0.0088 | 0.0032 | 23.4 | D | D | D | 5.31 |
| 27 | male | Ectopia | 9 | 100617286 | *FOXE1* | c.G1090A:p.G364S | missense SNV | 0.001 | 0.0006 | 18.23 | T | D | D | 3.88 |
|  |  |  | 14 | 81558898 | *TSHR* | c.T491A:p.M164K | missense SNV | NA | NA | 27.9 | D | D | D | 5.28 |
|  |  |  | 14 | 81609426 | *TSHR* | c.A1024T:p.K342X | nonsense | NA | NA | 35 | NA | NA | D | NA |
| 28 | male | Ectopia | 9 | 3828312 | *GLIS3* | c.G2753A:p.R918H | missense SNV | 0.001 | 0.0005 | 27.4 | T | P | D | 5.92 |
| 29 | male | Ectopia | 15 | 45409472 | *DUOXA2* | c.C738G:p.Y246X | nonsense | NA | 0.0025 | 34 | NA | NA | D | NA |
| 30 | female | Ectopia | 7 | 107314782 | *SLC26A4* | c.G589A:p.G197R | missense SNV | NA | 0.0001 | 33 | D | D | D | 5.07 |
| 31 | female | Ectopia | 15 | 45409472 | *DUOXA2* | c.C738G:p.Y246X | nonsense | NA | 0.0025 | 34 | NA | NA | D | NA |
| 32 | male | Ectopia | 3 | 24231772 | *THRB* | c.G76A:p.D26N | missense SNV | NA | 0.0001 | 15.45 | D | B | N | 3.92 |
|  |  |  | 14 | 81609976 | *TSHR* | c.T1574C:p.F525S | missense SNV | 0.001 | 0.0017 | 24 | T | D | D | 5.46 |
|  |  |  | 15 | 45409472 | *DUOXA2* | c.C738G:p.Y246X | nonsense | NA | 0.0025 | 34 | NA | NA | D | NA |
| 33 | male | Ectopia | 15 | 45409472 | *DUOXA2* | c.C738G:p.Y246X | nonsense | NA | 0.0025 | 34 | NA | NA | D | NA |
|  |  |  | 8 | 134024144 | *TG* | exon36:c.6263-2A>G | splicing | NA | NA | 20.3 | NA | NA | D | 5.58 |
| 34 | male | Athyreosis | 8 | 134128945 | *TG* | c.A7847T:p.N2616I | missense SNV | 0.0024 | 0.0016 | 12.35 | D | B | P | NA |
| 35 | female | Ectopia | 15 | 45444175 | *DUOX1* | c.A3118C:p.N1040H | missense SNV | NA | NA | 20.2 | T | D | D | 4.17 |
| 36 | male | Hypoplasia | 14 | 81609751 | *TSHR* | c.G1349A:p.R450H | missense SNV | 0.001 | 0.0034 | 32 | D | D | D | 5.74 |
|  |  |  | 15 | 45391884 | *DUOX2* | c.G3391T:p.A1131S | missense SNV | NA | 0.0003 | 34 | D | D | D | 5.58 |
|  |  |  | 15 | 45397973 | *DUOX2* | c.G2202A:p.W734X | nonsense | NA | 0.0003 | 35 | NA | NA | A | 2.4 |
|  |  |  | 15 | 45409472 | *DUOXA2* | c.C738G:p.Y246X | nonsense | NA | 0.0025 | 34 | NA | NA | D | NA |
| 37 | female | Ectopia | 14 | 81609751 | *TSHR* | c.G1349A:p.R450H | missense SNV | 0.001 | 0.0034 | 32 | D | D | D | 5.74 |
| 38 | male | Athyreosis | 15 | 45409472 | *DUOXA2* | c.C738G:p.Y246X | nonsense | NA | 0.0025 | 34 | NA | NA | D | NA |
| 39 | male | Ectopia | 2 | 113994290 | *PAX8* | c.C786A:p.Y262X | nonsense | NA | NA | 38 | NA | NA | A | 2.07 |
|  |  |  | 9 | 100616789 | *FOXE1* | c.C593G:p.A198G | missense SNV | NA | NA | 23.3 | D | P | N | 3.03 |
| 40 | male | Athyreosis | 15 | 45391946 | *DUOX2* | c.G3329A:p.R1110Q | missense SNV | 0.002 | 0.0025 | 35 | D | D | D | 5.6 |
|  |  |  | 15 | 45412435 | *DUOXA1* | c.C503T:p.T168M | missense SNV | 0.002 | 0.0036 | 29.6 | D | D | D | 5.12 |
| 41 | female | Athyreosis | 15 | 45409472 | *DUOXA2* | c.C738G:p.Y246X | nonsense | NA | 0.0025 | 34 | NA | NA | D | NA |
|  |  |  | 8 | 134125846 | *TG* | c.C7753T:p.R2585W | missense SNV | 0.0069 | 0.0055 | 33 | D | D | D | NA |
| 42 | male | Athyreosis | 9 | 100616530 | *FOXE1* | c.C334T:p.L112F | missense SNV | NA | 0.0001 | 27.4 | D | D | D | 2.97 |
| 43 | male | Ectopia | 15 | 45454115 | *DUOX1* | c.C4036G:p.L1346V | missense SNV | NA | NA | 27.2 | D | D | D | 4.22 |
| 44 | male | Athyreosis | 15 | 45409472 | *DUOXA2* | c.C738G:p.Y246X | nonsense | NA | 0.0025 | 34 | NA | NA | D | NA |
| 45 | male | Athyreosis | 15 | 45396158 | *DUOX2* | c.G2654T:p.R885L | missense SNV | 0.004 | 0.0044 | 27.5 | D | D | D | 4.29 |
|  |  |  | 15 | 45403784 | *DUOX2* | exon6:c.514-1G>A | splicing | NA | NA | 26.3 | NA | NA | D | 5.06 |
|  |  |  | 15 | 45409472 | *DUOXA2* | c.C738G:p.Y246X | nonsense | NA | 0.0025 | 34 | NA | NA | D | NA |
|  |  |  | 8 | 133909932 | *TG* | c.G3040C:p.D1014H | missense SNV | 0.001 | 0.0008 | 10.2 | D | P | N | NA |
| 46 | male | Athyreosis | 15 | 45408396 | *DUOXA2* | c.C280T:p.R94C | missense SNV | NA | NA | 20.4 | T | D | N | 5.38 |
|  |  |  | 2 | 1481101 | *TPO* | c.C1063T:p.R355C | missense SNV | NA | NA | 21.1 | T | D | N | 3.12 |
| 47 | male | Athyreosis | 15 | 45402159 | *DUOX2* | c.C1060T:p.R354W | missense SNV | NA | 0.0001 | 34 | D | D | D | 4.82 |
|  |  |  | 15 | 45409472 | *DUOXA2* | c.C738G:p.Y246X | nonsense | NA | 0.0025 | 34 | NA | NA | D | NA |
| 48 | male | Athyreosis | 15 | 45444526 | *DUOX1* | c.C3236A:p.T1079N | missense SNV | NA | NA | 24.8 | D | D | D | 4.07 |
| 49 | female | Athyreosis | 15 | 45409472 | *DUOXA2* | c.C738G:p.Y246X | nonsense | NA | 0.0025 | 34 | NA | NA | D | NA |
|  |  |  | 8 | 134128945 | *TG* | c.A7847T:p.N2616I | missense SNV | 0.0024 | 0.0016 | 12.35 | D | B | P | NA |
| 50 | male | Athyreosis | 9 | 3828387 | *GLIS3* | c.C2678T:p.S893F | missense SNV | 0.003 | 0.0018 | 26.5 | D | D | D | 5.92 |
|  |  |  | 15 | 45396563 | *DUOX2* | c.G2335A:p.V779M | missense SNV | 0.005 | 0.0056 | 28.4 | D | P | D | 5.53 |
| 51 | male | Athyreosis | 6 | 150713627 | *IYD* | c.C517A:p.L173I(NM_001164695; NM_203395) | missense SNV | NA | NA | 24.9 | D | D | D | 2.04 |
| 52 | male | Athyreosis | 15 | 45409472 | *DUOXA2* | c.C738G:p.Y246X | nonsense | NA | 0.0025 | 34 | NA | NA | D | NA |
|  |  |  | 6 | 150716549 | *IYD* | c.T707C:p.L236P (NM_001164695) | missense SNV | 0.001 | NA | 10.05 | D | D | N | NA |
| 53 | male | Athyreosis | 15 | 45409472 | *DUOXA2* | c.C738G:p.Y246X | nonsense | NA | 0.0025 | 34 | NA | NA | D | NA |
| 54 | female | Hypoplasia | 15 | 45409472 | *DUOXA2* | c.C738G:p.Y246X | nonsense | NA | 0.0025 | 34 | NA | NA | D | NA |
| 55 | male | Athyreosis | 14 | 36986635 | *NKX2-1* | c.G964A:p.G322S | missense SNV | 0.003 | 0.0039 | 13.89 | T | B | D | 2.65 |
| 56 | female | Hypoplasia | 3 | 24185051 | *THRB* | c.G679A:p.E227K | missense SNV | NA | NA | 19.11 | T | P | D | 6.04 |
|  |  |  | 14 | 81557483 | *TSHR* | c.A463G:p.I155V | missense SNV | NA | 0.0001 | 16.74 | T | B | D | 4.59 |
| 57 | female | Athyreosis | 15 | 45408396 | *DUOXA2* | c.C280T:p.R94C | missense SNV | NA | NA | 20.4 | T | D | N | 5.38 |
|  |  |  | 15 | 45408819 | *DUOXA2* | c.G446A:p.G149E | missense SNV | NA | 0.0001 | 26.1 | D | D | D | 5.58 |
|  |  |  | 15 | 45440145 | *DUOX1* | c.C2592A:p.D864E | missense SNV | NA | NA | 32 | D | D | D | 4.45 |
| 58 | male | Athyreosis | 15 | 45396563 | *DUOX2* | c.G2335A:p.V779M | missense SNV | 0.005 | 0.0056 | 28.4 | D | P | D | 5.53 |
| 59 | male | Athyreosis | 14 | 81554378 | *TSHR* | exon4:c.392+6T>G | splicing | NA | NA | 15.24 | NA | NA | NA | 5.84 |
|  |  |  | 15 | 45396158 | *DUOX2* | c.G2654T:p.R885L | missense SNV | 0.004 | 0.0044 | 27.5 | D | D | D | 4.29 |
|  |  |  | 7 | 107323898 | *SLC26A4* | exon8:c.919-2A>G | splicing | NA | 0.0043 | 24.8 | NA | NA | D | 5.62 |
| 60 | female | Hypoplasia | 15 | 45393403 | *DUOX2* | c.G2921A:p.R974H | missense SNV | NA | 0.0015 | 22.3 | D | B | D | 3.98 |
|  |  |  | 8 | 134107420 | *TG* | c.C7372T:p.P2458S | missense SNV | 0.001 | 0.0013 | 21.1 | T | D | D | 6.07 |
| 61 | male | Hypoplasia | 15 | 45398370 | *DUOX2* | c.C2101T:p.R701X | nonsense | NA | 0.0001 | 36 | NA | NA | A | 2.36 |
| 62 | female | Hypoplasia | 15 | 45397885 | *DUOX2* | c.C2290T:p.R764W | missense SNV | NA | 0 | 34 | D | D | D | 3.67 |
|  |  |  | 8 | 133981767 | *TG* | c.A5928G:p.I1976M | missense SNV | NA | 0 | 41 | D | P | N | NA |
|  |  |  | 2 | 1481120 | *TPO* | c.G1082T:p.R361L | missense SNV | 0.0188 | 0.0176 | 25.3 | T | P | D | 4.99 |
| 63 | female | Hypoplasia | 15 | 45388079 | *DUOX2* | c.C4027T:p.L1343F | missense SNV | 0.005 | 0.0068 | 29.5 | T | P | D | 5.57 |
|  |  |  | 15 | 45398423 | *DUOX2* | c.G2048T:p.R683L | missense SNV | 0.003 | 0.0038 | 34 | D | D | D | 5.31 |
|  |  |  | 15 | 45401075 | *DUOX2* | c.G1310C:p.G437A | missense SNV | NA | 0.0021 | 27.9 | D | D | D | 5.11 |
|  |  |  | 15 | 45411399 | *DUOXA1* | c.A802G:p.S268G | missense SNV | 0.0088 | 0.0032 | 23.4 | D | D | D | 5.31 |
| 64 | female | Hypoplasia | 14 | 36986635 | *NKX2-1* | c.G964A:p.G322S | missense SNV | 0.003 | 0.0039 | 13.89 | T | B | D | 2.65 |
|  |  |  | 15 | 45391946 | *DUOX2* | c.G3329A:p.R1110Q | missense SNV | 0.002 | 0.0025 | 35 | D | D | D | 5.6 |
|  |  |  | 15 | 45412435 | *DUOXA1* | c.C503T:p.T168M | missense SNV | 0.002 | 0.0036 | 29.6 | D | D | D | 5.12 |
| 65 | male | Ectopia | 2 | 1544412 | *TPO* | c.G2494A:p.G832R | missense SNV | NA | 0.0003 | 17.26 | D | D | N | NA |
| 66 | female | Athyreosis | 14 | 81609984 | *TSHR* | c.C1582A:p.R528S | missense SNV | NA | NA | 25.3 | D | B | D | 5.46 |
| 67 | male | Athyreosis | 14 | 36986583 | *NKX2-1* | c.C1016T:p.A339V | missense SNV | 0.002 | 0.0036 | 21.9 | T | P | N | 3.07 |
|  |  |  | 15 | 45389484 | *DUOX2* | c.C3799T:p.R1267W | missense SNV | NA | 0 | 34 | D | D | D | 4.15 |
| 68 | male | Athyreosis | 15 | 45393403 | *DUOX2* | c.G2921A:p.R974H | missense SNV | NA | 0.0015 | 22.3 | D | B | D | 3.98 |
|  |  |  | 7 | 107330644 | *SLC26A4* | c.C1225T:p.R409C | missense SNV | NA | 0.0001 | 35 | D | D | D | 5.1 |
| 69 | female | Ectopia | 15 | 45391946 | *DUOX2* | c.G3329A:p.R1110Q | missense SNV | 0.002 | 0.0025 | 35 | D | D | D | 5.6 |
|  |  |  | 15 | 45412435 | *DUOXA1* | c.C503T:p.T168M | missense SNV | 0.002 | 0.0036 | 29.6 | D | D | D | 5.12 |
| 70 | female | Ectopia | 15 | 45386877 | *DUOX2* | c.C4408T:p.R1470W | missense SNV | 0.004 | 0.0019 | 35 | D | D | D | 4.74 |
|  |  |  | 15 | 45409472 | *DUOXA2* | c.C738G:p.Y246X | nonsense | NA | 0.0025 | 34 | NA | NA | D | NA |
| 71 | female | Gland-in-Situ | 15 | 45409472 | *DUOXA2* | c.C738G:p.Y246X | nonsense | NA | 0.0025 | 34 | NA | NA | D | NA |
| 72 | male | Gland-in-Situ | 15 | 45409472 | *DUOXA2* | c.C738G:p.Y246X | nonsense | NA | 0.0025 | 34 | NA | NA | D | NA |
| 73 | male | Gland-in-Situ | 15 | 45409472 | *DUOXA2* | c.C738G:p.Y246X | nonsense | NA | 0.0025 | 34 | NA | NA | D | NA |
| 74 | female | Ectopia | 15 | 45409472 | *DUOXA2* | c.C738G:p.Y246X | nonsense | NA | 0.0025 | 34 | NA | NA | D | NA |
| 75 | male | Ectopia | 15 | 45396158 | *DUOX2* | c.G2654T:p.R885L | missense SNV | 0.004 | 0.0044 | 27.5 | D | D | D | 4.29 |
| 76 | male | Ectopia | 15 | 45396563 | *DUOX2* | c.G2335A:p.V779M | missense SNV | 0.005 | 0.0056 | 28.4 | D | P | D | 5.53 |
|  |  |  | 19 | 17992872 | *SLC5A5* | c.A1162T:p.K388X | nonsense | NA | NA | 42 | NA | NA | A | 4.36 |
| 77 | female | Hypoplasia | 15 | 45391884 | *DUOX2* | c.G3391T:p.A1131S | missense SNV | NA | 0.0003 | 34 | D | D | D | 5.58 |
|  |  |  | 15 | 45397973 | *DUOX2* | c.G2202A:p.W734X | nonsense | NA | 0.0003 | 35 | NA | NA | A | 2.4 |
|  |  |  | 15 | 45409472 | *DUOXA2* | c.C738G:p.Y246X | nonsense | NA | 0.0025 | 34 | NA | NA | D | NA |
| 78 | female | Hypoplasia | 14 | 81609751 | *TSHR* | c.G1349A:p.R450H | missense SNV | 0.001 | 0.0034 | 32 | D | D | D | 5.74 |
| 79 | female | Gland-in-Situ | 8 | 134125846 | *TG* | c.C7753T:p.R2585W | missense SNV | 0.0069 | 0.0055 | 33 | D | D | D | NA |
| 80 | female | Ectopia | 8 | 133925442 | *TG* | c.G4310A:p.W1437X | nonsense | NA | NA | 35 | NA | NA | A | NA |
| 81 | male | Ectopia | 9 | 100616953 | *FOXE1* | c.G757A:p.G253S | missense SNV | NA | 0 | 10.6 | D | P | N | 2.94 |
|  |  |  | 15 | 45409472 | *DUOXA2* | c.C738G:p.Y246X | nonsense | NA | 0.0025 | 34 | NA | NA | D | NA |
| 82 | male | Hypoplasia | 8 | 134128945 | *TG* | c.A7847T:p.N2616I | missense SNV | 0.0024 | 0.0016 | 12.35 | D | B | P | NA |
| 83 | female | Hypoplasia | 15 | 45436386 | *DUOX1* | c.C2089T:p.R697C | missense SNV | NA | 0.0001 | 24.2 | T | B | D | 4.78 |
| 84 | female | Hypoplasia | 15 | 45409472 | *DUOXA2* | c.C738G:p.Y246X | nonsense | NA | 0.0025 | 34 | NA | NA | D | NA |
| 85 | male | Hypoplasia | 7 | 107323898 | *SLC26A4* | exon8:c.919-2A>G | splicing | NA | 0.0043 | 24.8 | NA | NA | D | 5.62 |
| 86 | male | Gland-in-Situ | 2 | 1481120 | *TPO* | c.G1082T:p.R361L | missense SNV | 0.0188 | 0.0176 | 25.3 | T | P | D | 4.99 |
| 87 | female | Gland-in-Situ | 15 | 45398798 | *DUOX2* | c.C1873T:p.R625X | nonsense | NA | 0 | 38 | NA | NA | A | 4.42 |
|  |  |  | 8 | 133948050 | *TG* | c.G4982A:p.R1661H | missense SNV | NA | 0.0015 | NA | D | D | N | NA |
| 88 | male | Hypoplasia | 2 | 1481120 | *TPO* | c.G1082T:p.R361L | missense SNV | 0.0188 | 0.0176 | 25.3 | T | P | D | 4.99 |
| 89 | male | Hypoplasia | 15 | 45388079 | *DUOX2* | c.C4027T:p.L1343F | missense SNV | 0.005 | 0.0068 | 29.5 | T | P | D | 5.57 |
|  |  |  | 15 | 45398423 | *DUOX2* | c.G2048T:p.R683L | missense SNV | 0.003 | 0.0038 | 34 | D | D | D | 5.31 |
|  |  |  | 15 | 45401724 | *DUOX2* | c.G1232A:p.R411K | missense SNV | NA | 0.0007 | 29.6 | D | B | D | 5.36 |
|  |  |  | 15 | 45411399 | *DUOXA1* | c.A802G:p.S268G | missense SNV | 0.0088 | 0.0032 | 23.4 | D | D | D | 5.31 |
|  |  |  | 8 | 133910471 | *TG* | c.G3197A:p.R1066H | missense SNV | 0.005 | 0.004 | 24.4 | D | D | D | 4.03 |
| 90 | male | Gland-in-Situ | 15 | 45396177 | *DUOX2* | c.G2635A:p.E879K | missense SNV | NA | 0.0006 | 34 | D | D | D | 5.23 |
|  |  |  | 15 | 45403623 | *DUOX2* | c.C674T:p.P225L | missense SNV | NA | NA | 23.4 | T | P | D | 5.06 |
| 91 | male | Gland-in-Situ | 14 | 36986635 | *NKX2-1* | c.G964A:p.G322S | missense SNV | 0.003 | 0.0039 | 13.89 | T | B | D | 2.65 |
|  |  |  | 14 | 81609976 | *TSHR* | c.T1574C:p.F525S | missense SNV | 0.001 | 0.0017 | 24 | T | D | D | 5.46 |
|  |  |  | 15 | 45409472 | *DUOXA2* | c.C738G:p.Y246X | nonsense | NA | 0.0025 | 34 | NA | NA | D | NA |
|  |  |  | 8 | 133919106 | *TG* | c.C3808T:p.R1270C | missense SNV | NA | 0.0024 | 29.2 | D | D | N | 4.51 |
| 92 | female | Gland-in-Situ | 15 | 45387181 | *DUOX2* | c.T4348C:p.Y1450H | missense SNV | NA | 0.0002 | 28 | D | D | D | 5.54 |
| 93 | male | Gland-in-Situ | 14 | 81609775 | *TSHR* | c.T1373A:p.F458Y | missense SNV | NA | NA | 27.4 | D | D | D | 5.74 |
|  |  |  | 15 | 45409472 | *DUOXA2* | c.C738G:p.Y246X | nonsense | NA | 0.0025 | 34 | NA | NA | D | NA |
|  |  |  | 8 | 134107420 | *TG* | c.C7372T:p.P2458S | missense SNV | 0.001 | 0.0013 | 21.1 | T | D | D | 6.07 |
| 94 | male | Gland-in-Situ | 15 | 45391946 | *DUOX2* | c.G3329A:p.R1110Q | missense SNV | 0.002 | 0.0025 | 35 | D | D | D | 5.6 |
|  |  |  | 15 | 45398798 | *DUOX2* | c.C1873T:p.R625X | nonsense | NA | 0 | 38 | NA | NA | A | 4.42 |
|  |  |  | 15 | 45412435 | *DUOXA1* | c.C503T:p.T168M | missense SNV | 0.002 | 0.0036 | 29.6 | D | D | D | 5.12 |
| 95 | female | Gland-in-Situ | 15 | 45409307 | *DUOXA2* | c.G573A:p.W191X | nonsense | NA | NA | 41 | NA | NA | A | 4.06 |
| 96 | male | Gland-in-Situ | 15 | 45391946 | *DUOX2* | c.G3329A:p.R1110Q | missense SNV | 0.002 | 0.0025 | 35 | D | D | D | 5.6 |
|  |  |  | 15 | 45412435 | *DUOXA1* | c.C503T:p.T168M | missense SNV | 0.002 | 0.0036 | 29.6 | D | D | D | 5.12 |
| 97 | male | Gland-in-Situ | 15 | 45396158 | *DUOX2* | c.G2654T:p.R885L | missense SNV | 0.004 | 0.0044 | 27.5 | D | D | D | 4.29 |
| 98 | female | Gland-in-Situ | 15 | 45386428 | *DUOX2* | c.A4567G:p.T1523A | missense SNV | 0.001 | 0.0001 | 27.6 | D | D | D | 5.36 |
| 99 | female | Hypoplasia | 9 | 3856011 | *GLIS3* | c.A2471G:p.H824R | missense SNV | 0.005 | 0.0071 | 23.1 | D | P | D | 5.8 |
| 100 | male | Gland-in-Situ | 15 | 45388079 | *DUOX2* | c.C4027T:p.L1343F | missense SNV | 0.005 | 0.0068 | 29.5 | T | P | D | 5.57 |
|  |  |  | 15 | 45411399 | *DUOXA1* | c.A802G:p.S268G | missense SNV | 0.0088 | 0.0032 | 23.4 | D | D | D | 5.31 |
| 101 | male | Hypoplasia | 15 | 45396563 | *DUOX2* | c.G2335A:p.V779M | missense SNV | 0.005 | 0.0056 | 28.4 | D | P | D | 5.53 |
| 102 | female | Gland-in-Situ | 15 | 45401724 | *DUOX2* | c.G1232A:p.R411K | missense SNV | NA | 0.0007 | 29.6 | D | B | D | 5.36 |
|  |  |  | 2 | 1520672 | *TPO* | c.C2365T:p.R789W | missense SNV | 0.002 | 0.0017 | 25.2 | D | D | N | 2.1 |
| 103 | female | Gland-in-Situ | 15 | 45409472 | *DUOXA2* | c.C738G:p.Y246X | nonsense | NA | 0.0025 | 34 | NA | NA | D | NA |
| 104 | male | Athyreosis | 15 | 45388079 | *DUOX2* | c.C4027T:p.L1343F | missense SNV | 0.005 | 0.0068 | 29.5 | T | P | D | 5.57 |
|  |  |  | 15 | 45411399 | *DUOXA1* | c.A802G:p.S268G | missense SNV | 0.0088 | 0.0032 | 23.4 | D | D | D | 5.31 |
| 105 | female | Gland-in-Situ | 15 | 45409472 | *DUOXA2* | c.C738G:p.Y246X | nonsense | NA | 0.0025 | 34 | NA | NA | D | NA |
| 106 | female | Hypoplasia | 9 | 100616530 | *FOXE1* | c.C334T:p.L112F | missense SNV | NA | 0.0001 | 27.4 | D | D | D | 2.97 |
|  |  |  | 15 | 45392024 | *DUOX2* | c.G3251A:p.R1084Q | missense SNV | NA | 0.0002 | 34 | D | D | D | 5.6 |
|  |  |  | 15 | 45409472 | *DUOXA2* | c.C738G:p.Y246X | nonsense | NA | 0.0025 | 34 | NA | NA | D | NA |
|  |  |  | 8 | 133899061 | *TG* | c.C1444T:p.Q482X | nonsense | NA | NA | 36 | NA | NA | A | 4.76 |
| 107 | female | Gland-in-Situ | 9 | 3879532 | *GLIS3* | c.C2192G:p.P731R | missense SNV | NA | NA | 26.4 | D | D | D | 5.98 |
|  |  |  | 15 | 45391946 | *DUOX2* | c.G3329A:p.R1110Q | missense SNV | 0.002 | 0.0025 | 35 | D | D | D | 5.6 |
|  |  |  | 15 | 45409472 | *DUOXA2* | c.C738G:p.Y246X | nonsense | NA | 0.0025 | 34 | NA | NA | D | NA |
|  |  |  | 15 | 45412435 | *DUOXA1* | c.C503T:p.T168M | missense SNV | 0.002 | 0.0036 | 29.6 | D | D | D | 5.12 |
| 108 | male | Hypoplasia | 14 | 81557414 | *TSHR* | c.G394C:p.G132R | missense SNV | NA | 0.0005 | 23.8 | T | D | D | 5.8 |
|  |  |  | 14 | 81610468 | *TSHR* | c.T2066G:p.V689G | missense SNV | NA | 0.0006 | 26 | D | D | D | 5.23 |
| 109 | male | Gland-in-Situ | 14 | 36986635 | *NKX2-1* | c.G964A:p.G322S | missense SNV | 0.003 | 0.0039 | 13.89 | T | B | D | 2.65 |
| 110 | female | Gland-in-Situ | 15 | 45388079 | *DUOX2* | c.C4027T:p.L1343F | missense SNV | 0.005 | 0.0068 | 29.5 | T | P | D | 5.57 |
|  |  |  | 15 | 45398423 | *DUOX2* | c.G2048T:p.R683L | missense SNV | 0.003 | 0.0038 | 34 | D | D | D | 5.31 |
|  |  |  | 15 | 45411399 | *DUOXA1* | c.A802G:p.S268G | missense SNV | 0.0088 | 0.0032 | 23.4 | D | D | D | 5.31 |
| 111 | female | Gland-in-Situ | 15 | 45396563 | *DUOX2* | c.G2335A:p.V779M | missense SNV | 0.005 | 0.0056 | 28.4 | D | P | D | 5.53 |
| 112 | male | Athyreosis | 8 | 134125826 | *TG* | c.G7733A:p.R2578Q | missense SNV | 0.001 | 0.0002 | 24.4 | D | D | N | 3.91 |
| 113 | male | Gland-in-Situ | 15 | 45404115 | *DUOX2* | c.C364A:p.P122T | missense SNV | 0.002 | 0.0002 | 24.2 | D | B | D | 4.3 |
|  |  |  | 15 | 45409472 | *DUOXA2* | c.C738G:p.Y246X | nonsense | NA | 0.0025 | 34 | NA | NA | D | NA |
|  |  |  | 15 | 45457004 | *DUOX1* | c.G4561A:p.G1521S | missense SNV | NA | NA | 33 | D | D | D | 4.47 |
| 114 | male | Gland-in-Situ | 15 | 45409472 | *DUOXA2* | c.C738G:p.Y246X | nonsense | NA | 0.0025 | 34 | NA | NA | D | NA |
| 115 | female | Gland-in-Situ | 15 | 45398798 | *DUOX2* | c.C1873T:p.R625X | nonsense | NA | 0 | 38 | NA | NA | A | 4.42 |
| 116 | male | Gland-in-Situ | 15 | 45442895 | *DUOX1* | c.A2884T:p.I962F | missense SNV | NA | 0.0001 | NA | T | P | D | NA |
| 117 | female | Hypoplasia | 15 | 45409472 | *DUOXA2* | c.C738G:p.Y246X | nonsense | NA | 0.0025 | 34 | NA | NA | D | NA |
| 118 | male | Hypoplasia | 14 | 81563048 | *TSHR* | c.C611T:p.A204V | missense SNV | NA | 0.0002 | 27.1 | T | D | D | 6.02 |
| 119 | male | Hypoplasia | 2 | 114002108 | *PAX8* | c.C285G:p.Y95X | nonsense | NA | NA | 38 | NA | NA | A | 5.32 |
|  |  |  | 9 | 4118540 | *GLIS3* | c.G938C:p.G313A | missense SNV | 0.004 | 0.0024 | 25.6 | D | D | D | 5.59 |
|  |  |  | 14 | 81609640 | *TSHR* | c.G1238A:p.G413D | missense SNV | NA | NA | 27.8 | D | D | D | 5.88 |
|  |  |  | 15 | 45386880 | *DUOX2* | c.G4405A:p.E1469K | missense SNV | NA | 0 | 35 | D | D | D | 5.68 |
| 120 | male | Athyreosis | 15 | 45409472 | *DUOXA2* | c.C738G:p.Y246X | nonsense | NA | 0.0025 | 34 | NA | NA | D | NA |
| 121 | female | Hypoplasia | 15 | 45412435 | *DUOXA1* | c.C503T:p.T168M | missense SNV | 0.002 | 0.0036 | 29.6 | D | D | D | 5.12 |
| 122 | female | Athyreosis | 8 | 134128945 | *TG* | c.A7847T:p.N2616I | missense SNV | 0.0024 | 0.0016 | 12.35 | D | B | P | NA |
| 123 | male | Gland-in-Situ | 14 | 36986635 | *NKX2-1* | c.G964A:p.G322S | missense SNV | 0.003 | 0.0039 | 13.89 | T | B | D | 2.65 |
| 124 | female | Gland-in-Situ | 15 | 45409472 | *DUOXA2* | c.C738G:p.Y246X | nonsense | NA | 0.0025 | 34 | NA | NA | D | NA |
| 125 | female | Gland-in-Situ | 15 | 45409472 | *DUOXA2* | c.C738G:p.Y246X | nonsense | NA | 0.0025 | 34 | NA | NA | D | NA |
| 126 | female | Hypoplasia | 14 | 36986583 | *NKX2-1* | c.C1016T:p.A339V | missense SNV | 0.002 | 0.0036 | 21.9 | T | P | N | 3.07 |
|  |  |  | 15 | 45388079 | *DUOX2* | c.C4027T:p.L1343F | missense SNV | 0.005 | 0.0068 | 29.5 | T | P | D | 5.57 |
|  |  |  | 15 | 45411399 | *DUOXA1* | c.A802G:p.S268G | missense SNV | 0.0088 | 0.0032 | 23.4 | D | D | D | 5.31 |
| 127 | male | Gland-in-Situ | 7 | 107330645 | *SLC26A4* | c.G1226A:p.R409H | missense SNV | NA | 0 | 34 | D | D | D | 5.1 |
| 128 | female | Hypoplasia | 5 | 172659699 | *NKX2-5* | c.C848A:p.P283Q | missense SNV | NA | 0.0005 | 11.39 | T | B | D | 3.39 |
|  |  |  | 15 | 45409472 | *DUOXA2* | c.C738G:p.Y246X | nonsense | NA | 0.0025 | 34 | NA | NA | D | NA |
| 129 | female | Hypoplasia | 15 | 45388079 | *DUOX2* | c.C4027T:p.L1343F | missense SNV | 0.005 | 0.0068 | 29.5 | T | P | D | 5.57 |
|  |  |  | 15 | 45398423 | *DUOX2* | c.G2048T:p.R683L | missense SNV | 0.003 | 0.0038 | 34 | D | D | D | 5.31 |
|  |  |  | 15 | 45411399 | *DUOXA1* | c.A802G:p.S268G | missense SNV | 0.0088 | 0.0032 | 23.4 | D | D | D | 5.31 |
|  |  |  | 2 | 1499831 | *TPO* | c.C1906T:p.R636W | missense SNV | NA | 0.0001 | 34 | D | D | A | 3.58 |
| 130 | female | Hypoplasia | 15 | 45386458 | *DUOX2* | c.G4537C:p.G1513R | missense SNV | NA | NA | 34 | D | D | D | 5.36 |
|  |  |  | 15 | 45409472 | *DUOXA2* | c.C738G:p.Y246X | nonsense | NA | 0.0025 | 34 | NA | NA | D | NA |
| 131 | male | Hypoplasia | 2 | 1497754 | *TPO* | c.G1949A:p.G650E | missense SNV | 0.001 | 0.0001 | 25.2 | D | D | D | 4.84 |
| 132 | male | Gland-in-Situ | 15 | 45409472 | *DUOXA2* | c.C738G:p.Y246X | nonsense | NA | 0.0025 | 34 | NA | NA | D | NA |
|  |  |  | 2 | 1426892 | *TPO* | c.C170T:p.T57M | missense SNV | NA | NA | 24.7 | D | D | N | 3.72 |
|  |  |  | 2 | 1457597 | *TPO* | exon5:c.612+2T>G | splicing | NA | NA | 23.8 | NA | NA | D | 5.27 |
| 133 | male | Gland-in-Situ | 15 | 45404850 | *DUOX2* | c.C227T:p.P76L | missense SNV | NA | 0.0005 | 35 | D | D | D | 5.84 |
| 134 | female | Gland-in-Situ | 9 | 100617170 | *FOXE1* | c.A974C:p.Y325S | missense SNV | NA | 0.0004 | 25.7 | D | D | D | 4.57 |
|  |  |  | 8 | 133910424 | *TG* | c.G3150A:p.W1050X | nonsense | NA | NA | 37 | NA | NA | A | 4.91 |
| 135 | female | Hypoplasia | 2 | 1544412 | *TPO* | c.G2494A:p.G832R | missense SNV | NA | 0.0003 | 17.26 | D | D | N | NA |
| 136 | male | Hypoplasia | 14 | 81609751 | *TSHR* | c.G1349A:p.R450H | missense SNV | 0.001 | 0.0034 | 32 | D | D | D | 5.74 |
| 137 | female | Hypoplasia | 8 | 133880359 | *TG* | exon2:c.68-1G>A | splicing | NA | NA | 24.9 | NA | NA | D | 5.38 |
|  |  |  | 8 | 133984055 | *TG* | c.C5992T:p.R1998X | nonsense | NA | 0 | 40 | NA | NA | A | 4.64 |
| 138 | male | Hypoplasia | 14 | 81606153 | *TSHR* | c.G823A:p.A275T | missense SNV | 0.001 | 0.0001 | 26.1 | D | D | D | 5.46 |
| 139 | male | Gland-in-Situ | 15 | 45409472 | *DUOXA2* | c.C738G:p.Y246X | nonsense | NA | 0.0025 | 34 | NA | NA | D | NA |
|  |  |  | 6 | 150719321 | *IYD* | c.C818T:p.T273M(NM_203395) | missense SNV | NA | 0.0009 | 15.53 | T | B | D | 4.18 |
| 140 | female | Gland-in-Situ | 15 | 45396177 | *DUOX2* | c.G2635A:p.E879K | missense SNV | NA | 0.0006 | 34 | D | D | D | 5.23 |
|  |  |  | 15 | 45409472 | *DUOXA2* | c.C738G:p.Y246X | nonsense | NA | 0.0025 | 34 | NA | NA | D | NA |
|  |  |  | 8 | 133900411 | *TG* | c.C2359T:p.R787X | nonsense | NA | 0 | 35 | NA | NA | A | 2.95 |
| 141 | male | Gland-in-Situ | 15 | 45388243 | *DUOX2* | c.A3863C:p.Q1288P | missense SNV | NA | NA | 22.7 | T | B | D | NA |
|  |  |  | 15 | 45391884 | *DUOX2* | c.G3391T:p.A1131S | missense SNV | NA | 0.0003 | 34 | D | D | D | 5.58 |
|  |  |  | 15 | 45397973 | *DUOX2* | c.G2202A:p.W734X | nonsense | NA | 0.0003 | 35 | NA | NA | A | 2.4 |
|  |  |  | 15 | 45409472 | *DUOXA2* | c.C738G:p.Y246X | nonsense | NA | 0.0025 | 34 | NA | NA | D | NA |
| 142 | male | Gland-in-Situ | 15 | 45408819 | *DUOXA2* | c.G446A:p.G149E | missense SNV | NA | 0.0001 | 26.1 | D | D | D | 5.58 |
|  |  |  | 15 | 45409472 | *DUOXA2* | c.C738G:p.Y246X | nonsense | NA | 0.0025 | 34 | NA | NA | D | NA |
| 143 | female | Gland-in-Situ | 15 | 45409472 | *DUOXA2* | c.C738G:p.Y246X | nonsense | NA | 0.0025 | 34 | NA | NA | D | NA |
| 144 | female | Gland-in-Situ | 14 | 81609672 | *TSHR* | c.G1270T:p.V424F | missense SNV | NA | 0 | 28.4 | D | D | D | 5.74 |
| 145 | male | Hypoplasia | 14 | 81609426 | *TSHR* | c.A1024T:p.K342X | nonsense | NA | NA | 35 | NA | NA | D | NA |
|  |  |  | 14 | 81610468 | *TSHR* | c.T2066G:p.V689G | missense SNV | NA | 0.0006 | 26 | D | D | D | 5.23 |
|  |  |  | 15 | 45409472 | *DUOXA2* | c.C738G:p.Y246X | nonsense | NA | 0.0025 | 34 | NA | NA | D | NA |
|  |  |  | 8 | 133923622 | *TG* | c.G4003A:p.V1335M | missense SNV | NA | NA | 24.9 | D | D | D | 5.51 |
| 146 | female | Hypoplasia | 14 | 81558873 | *TSHR* | exon6:c.468-2A>G | splicing | NA | 0.0001 | 24.8 | NA | NA | D | 5.28 |
| 147 | female | Athyreosis | 8 | 133984042 | *TG* | c.C5979A:p.F1993L | missense SNV | NA | NA | 32 | D | D | D | 4.67 |
| 148 | male | Athyreosis | 15 | 45398798 | *DUOX2* | c.C1873T:p.R625X | nonsense | NA | 0 | 38 | NA | NA | A | 4.42 |
|  |  |  | 15 | 45406818 | *DUOXA2* | c.C15A:p.N5K | missense SNV | NA | 0.0001 | 24.1 | T | P | N | NA |
|  |  |  | 8 | 133900333 | *TG* | c.C2281T:p.P761S | missense SNV | 0.001 | 0.0001 | 23.3 | D | D | D | 5.81 |
|  |  |  | 8 | 134128945 | *TG* | c.A7847T:p.N2616I | missense SNV | 0.0024 | 0.0016 | 12.35 | D | B | P | NA |
| 149 | male | Gland-in-Situ | 8 | 133919133 | *TG* | c.C3835T:p.R1279W | missense SNV | NA | 0.0001 | 23 | D | D | N | NA |
| 150 | female | Gland-in-Situ | 14 | 36986635 | *NKX2-1* | c.G964A:p.G322S | missense SNV | 0.003 | 0.0039 | 13.89 | T | B | D | 2.65 |
|  |  |  | 15 | 45396158 | *DUOX2* | c.G2654T:p.R885L | missense SNV | 0.004 | 0.0044 | 27.5 | D | D | D | 4.29 |
|  |  |  | 15 | 45402159 | *DUOX2* | c.C1060T:p.R354W | missense SNV | NA | 0.0001 | 34 | D | D | D | 4.82 |
| 151 | female | Athyreosis | 8 | 134107412 | *TG* | c.G7364A:p.R2455H | missense SNV | 0.005 | 0.0092 | 31 | D | D | D | 5.17 |
| 152 | female | Gland-in-Situ | 15 | 45398798 | *DUOX2* | c.C1873T:p.R625X | nonsense | NA | 0 | 38 | NA | NA | A | 4.42 |
|  |  |  | 15 | 45404850 | *DUOX2* | c.C227T:p.P76L | missense SNV | NA | 0.0005 | 35 | D | D | D | 5.84 |
| 153 | female | Gland-in-Situ | 15 | 45386428 | *DUOX2* | c.A4567G:p.T1523A | missense SNV | 0.001 | 0.0001 | 27.6 | D | D | D | 5.36 |
|  |  |  | 15 | 45391946 | *DUOX2* | c.G3329A:p.R1110Q | missense SNV | 0.002 | 0.0025 | 35 | D | D | D | 5.6 |
|  |  |  | 15 | 45412435 | *DUOXA1* | c.C503T:p.T168M | missense SNV | 0.002 | 0.0036 | 29.6 | D | D | D | 5.12 |
| 154 | female | Gland-in-Situ | 15 | 45391946 | *DUOX2* | c.G3329A:p.R1110Q | missense SNV | 0.002 | 0.0025 | 35 | D | D | D | 5.6 |
|  |  |  | 15 | 45399153 | *DUOX2* | c.C1708T:p.Q570X | nonsense | NA | NA | 38 | NA | NA | A | 4.99 |
|  |  |  | 15 | 45412435 | *DUOXA1* | c.C503T:p.T168M | missense SNV | 0.002 | 0.0036 | 29.6 | D | D | D | 5.12 |
|  |  |  | 8 | 133919106 | *TG* | c.C3808T:p.R1270C | missense SNV | NA | 0.0024 | 29.2 | D | D | N | 4.51 |
| 155 | female | Hypoplasia | 15 | 45408819 | *DUOXA2* | c.G446A:p.G149E | missense SNV | NA | 0.0001 | 26.1 | D | D | D | 5.58 |
| 156 | male | Ectopia | 8 | 133894843 | *TG* | c.C875T:p.S292F | missense SNV | NA | NA | 25.7 | D | D | N | 5.63 |
| 157 | male | Hypoplasia | 15 | 45393424 | *DUOX2* | c.T2900C:p.I967T | missense SNV | NA | 0 | 23.4 | D | B | D | 4.91 |
|  |  |  | 15 | 45409472 | *DUOXA2* | c.C738G:p.Y246X | nonsense | NA | 0.0025 | 34 | NA | NA | D | NA |
| 158 | male | Athyreosis | 15 | 45402165 | *DUOX2* | c.C1054T:p.H352Y | missense SNV | NA | NA | 24.5 | D | P | D | 4.82 |
| 159 | male | Athyreosis | 5 | 172659675 | *NKX2-5* | c.A872T:p.N291I | missense SNV | NA | NA | 27.1 | D | P | D | 2.97 |
|  |  |  | 14 | 81609751 | *TSHR* | c.G1349A:p.R450H | missense SNV | 0.001 | 0.0034 | 32 | D | D | D | 5.74 |
| 160 | male | Athyreosis | 15 | 45388060 | *DUOX2* | c.C4046G:p.S1349C | missense SNV | NA | 0.001 | 10.61 | D | B | N | NA |
| 161 | male | Ectopia | 15 | 45396517 | *DUOX2* | c.C2381T:p.S794F | missense SNV | NA | NA | 26.7 | D | D | D | 5.53 |
|  |  |  | 15 | 45409472 | *DUOXA2* | c.C738G:p.Y246X | nonsense | NA | 0.0025 | 34 | NA | NA | D | NA |
| 162 | male | Ectopia | 15 | 45409472 | *DUOXA2* | c.C738G:p.Y246X | nonsense | NA | 0.0025 | 34 | NA | NA | D | NA |
| 163 | female | Gland-in-Situ | 15 | 45396158 | *DUOX2* | c.G2654T:p.R885L | missense SNV | 0.004 | 0.0044 | 27.5 | D | D | D | 4.29 |
| 164 | male | Hypoplasia | 2 | 113999158 | *PAX8* | c.T747G:p.Y249X | nonsense | NA | NA | 34 | NA | NA | A | NA |
|  |  |  | 15 | 45409472 | *DUOXA2* | c.C738G:p.Y246X | nonsense | NA | 0.0025 | 34 | NA | NA | D | NA |
| 165 | female | Gland-in-Situ | 15 | 45389889 | *DUOX2* | c.G3616A:p.A1206T | missense SNV | NA | 0.0003 | 34 | D | D | D | 5.69 |
|  |  |  | 15 | 45391946 | *DUOX2* | c.G3329A:p.R1110Q | missense SNV | 0.002 | 0.0025 | 35 | D | D | D | 5.6 |
|  |  |  | 15 | 45412435 | *DUOXA1* | c.C503T:p.T168M | missense SNV | 0.002 | 0.0036 | 29.6 | D | D | D | 5.12 |
| 166 | female | Gland-in-Situ | 15 | 45391946 | *DUOX2* | c.G3329A:p.R1110Q | missense SNV | 0.002 | 0.0025 | 35 | D | D | D | 5.6 |
|  |  |  | 15 | 45412435 | *DUOXA1* | c.C503T:p.T168M | missense SNV | 0.002 | 0.0036 | 29.6 | D | D | D | 5.12 |
|  |  |  | 8 | 133923770 | *TG* | c.A4151C:p.H1384P | missense SNV | NA | NA | 17.29 | D | P | N | NA |
|  |  |  | 2 | 1520714 | *TPO* | c.G2407A:p.G803R | missense SNV | NA | 0 | 22.9 | T | D | N | NA |
| 167 | male | Gland-in-Situ | 15 | 45396177 | *DUOX2* | c.G2635A:p.E879K | missense SNV | NA | 0.0006 | 34 | D | D | D | 5.23 |
|  |  |  | 15 | 45409472 | *DUOXA2* | c.C738G:p.Y246X | nonsense | NA | 0.0025 | 34 | NA | NA | D | NA |
| 168 | female | Athyreosis | 15 | 45391946 | *DUOX2* | c.G3329A:p.R1110Q | missense SNV | 0.002 | 0.0025 | 35 | D | D | D | 5.6 |
|  |  |  | 15 | 45412435 | *DUOXA1* | c.C503T:p.T168M | missense SNV | 0.002 | 0.0036 | 29.6 | D | D | D | 5.12 |
| 169 | male | Athyreosis | 15 | 45409472 | *DUOXA2* | c.C738G:p.Y246X | nonsense | NA | 0.0025 | 34 | NA | NA | D | NA |
| 170 | male | Athyreosis | 15 | 45396571 | *DUOX2* | exon19:c.2335-8G>A | splicing | 0.001 | 0.001 | 13 | NA | NA | NA | NA |
| 171 | male | Athyreosis | 6 | 150719321 | *IYD* | c.C818T:p.T273M(NM_203395) | missense SNV | NA | 0.0009 | 15.53 | T | B | D | 4.18 |
| 172 | male | Athyreosis | 19 | 17986789 | *SLC5A5* | c.A572G:p.D191G | missense SNV | NA | NA | 27.2 | D | D | D | 5 |
| 173 | female | Athyreosis | 15 | 45409472 | *DUOXA2* | c.C738G:p.Y246X | nonsense | NA | 0.0025 | 34 | NA | NA | D | NA |
| 174 | female | Ectopia | 15 | 45396563 | *DUOX2* | c.G2335A:p.V779M | missense SNV | 0.005 | 0.0056 | 28.4 | D | P | D | 5.53 |
| 175 | male | Ectopia | 15 | 45409472 | *DUOXA2* | c.C738G:p.Y246X | nonsense | NA | 0.0025 | 34 | NA | NA | D | NA |
|  |  |  | 2 | 1480873 | *TPO* | c.C835T:p.R279W | missense SNV | NA | 0.0008 | 24.3 | D | D | N | NA |
|  |  |  | 7 | 107302185 | *SLC26A4* | c.G99T:p.Q33H | missense SNV | NA | NA | 21.7 | D | P | D | 3.32 |
| 176 | male | Hypoplasia | 15 | 45408059 | *DUOXA2* | exon2:c.205+5G>A | splicing | NA | NA | 19.25 | NA | NA | NA | 5.58 |
|  |  |  | 15 | 45411399 | *DUOXA1* | c.A802G:p.S268G | missense SNV | 0.0088 | 0.0032 | 23.4 | D | D | D | 5.31 |
| 177 | female | Athyreosis | 8 | 133919106 | *TG* | c.C3808T:p.R1270C | missense SNV | NA | 0.0024 | 29.2 | D | D | N | 4.51 |
| 178 | male | Ectopia | 15 | 45403787 | *DUOX2* | exon6:c.514-4G>A | splicing | NA | 0 | 15.83 | NA | NA | NA | NA |
|  |  |  | 15 | 45411399 | *DUOXA1* | c.A802G:p.S268G | missense SNV | 0.0088 | 0.0032 | 23.4 | D | D | D | 5.31 |
| 179 | female | Athyreosis | 14 | 81609317 | *TSHR* | c.T915A:p.S305R | missense SNV | 0.006 | 0.003 | 11.51 | T | P | N | NA |
|  |  |  | 15 | 45411399 | *DUOXA1* | c.A802G:p.S268G | missense SNV | 0.0088 | 0.0032 | 23.4 | D | D | D | 5.31 |
|  |  |  | 8 | 134107412 | *TG* | c.G7364A:p.R2455H | missense SNV | 0.005 | 0.0092 | 31 | D | D | D | 5.17 |
| 180 | male | Athyreosis | 15 | 45397884 | *DUOX2* | c.G2291A:p.R764Q | missense SNV | 0.001 | 0.0002 | 35 | D | D | D | 5.63 |
| 181 | male | Ectopia | 15 | 45409472 | *DUOXA2* | c.C738G:p.Y246X | nonsense | NA | 0.0025 | 34 | NA | NA | D | NA |
| 182 | male | Ectopia | 14 | 81609976 | *TSHR* | c.T1574C:p.F525S | missense SNV | 0.001 | 0.0017 | 24 | T | D | D | 5.46 |
|  |  |  | 8 | 134128945 | *TG* | c.A7847T:p.N2616I | missense SNV | 0.0024 | 0.0016 | 12.35 | D | B | P | NA |
| 183 | male | Hypoplasia | 15 | 45409472 | *DUOXA2* | c.C738G:p.Y246X | nonsense | NA | 0.0025 | 34 | NA | NA | D | NA |
| 184 | male | Hypoplasia | 8 | 134107412 | *TG* | c.G7364A:p.R2455H | missense SNV | 0.005 | 0.0092 | 31 | D | D | D | 5.17 |
| 185 | male | Athyreosis | 9 | 3828319 | *GLIS3* | c.G2746A:p.V916M | missense SNV | NA | 0 | 23.6 | T | P | N | 3.11 |
| 186 | female | Ectopia | 15 | 45411399 | *DUOXA1* | c.A802G:p.S268G | missense SNV | 0.0088 | 0.0032 | 23.4 | D | D | D | 5.31 |
| 187 | male | Athyreosis | 15 | 45402140 | *DUOX2* | c.G1079T:p.G360V | missense SNV | NA | 0 | 15.21 | D | P | N | NA |
|  |  |  | 8 | 134125846 | *TG* | c.C7753T:p.R2585W | missense SNV | 0.0069 | 0.0055 | 33 | D | D | D | NA |
|  |  |  | 19 | 17992867 | *SLC5A5* | c.T1157G:p.I386S | missense SNV | NA | NA | 29.9 | D | D | D | 4.36 |
| 188 | female | Hypoplasia | 15 | 45409472 | *DUOXA2* | c.C738G:p.Y246X | nonsense | NA | 0.0025 | 34 | NA | NA | D | NA |
| 189 | male | Gland-in-Situ | 14 | 81557414 | *TSHR* | c.G394C:p.G132R | missense SNV | NA | 0.0005 | 23.8 | T | D | D | 5.8 |
|  |  |  | 14 | 81609751 | *TSHR* | c.G1349A:p.R450H | missense SNV | 0.001 | 0.0034 | 32 | D | D | D | 5.74 |
| 190 | male | Athyreosis | 8 | 134025848 | *TG* | c.G6401A:p.C2134Y | missense SNV | NA | 0 | 34 | D | D | D | 4.47 |
| 191 | male | Athyreosis | 2 | 113984778 | *PAX8* | c.C1064T:p.A355V | missense SNV | NA | 0 | 12.13 | T | B | D | NA |
|  |  |  | 14 | 81609751 | *TSHR* | c.G1349A:p.R450H | missense SNV | 0.001 | 0.0034 | 32 | D | D | D | 5.74 |
| 192 | male | Athyreosis | 14 | 81609751 | *TSHR* | c.G1349A:p.R450H | missense SNV | 0.001 | 0.0034 | 32 | D | D | D | 5.74 |
| 193 | male | Athyreosis | 15 | 45388079 | *DUOX2* | c.C4027T:p.L1343F | missense SNV | 0.005 | 0.0068 | 29.5 | T | P | D | 5.57 |
|  |  |  | 15 | 45394177 | *DUOX2* | c.G2665A:p.E889K | missense SNV | NA | NA | 26.2 | D | D | D | 5.69 |
|  |  |  | 15 | 45398423 | *DUOX2* | c.G2048T:p.R683L | missense SNV | 0.003 | 0.0038 | 34 | D | D | D | 5.31 |
|  |  |  | 15 | 45411399 | *DUOXA1* | c.A802G:p.S268G | missense SNV | 0.0088 | 0.0032 | 23.4 | D | D | D | 5.31 |
|  |  |  | 15 | 45412435 | *DUOXA1* | c.C503T:p.T168M | missense SNV | 0.002 | 0.0036 | 29.6 | D | D | D | 5.12 |
| 194 | male | Athyreosis | 15 | 45396158 | *DUOX2* | c.G2654T:p.R885L | missense SNV | 0.004 | 0.0044 | 27.5 | D | D | D | 4.29 |
| 195 | male | Athyreosis | 15 | 45397861 | *DUOX2* | c.T2314C:p.F772L | missense SNV | NA | NA | 25.8 | T | D | D | 5.63 |
|  |  |  | 15 | 45409472 | *DUOXA2* | c.C738G:p.Y246X | nonsense | NA | 0.0025 | 34 | NA | NA | D | NA |
| 196 | male | Athyreosis | 9 | 3829459 | *GLIS3* | c.C2507A:p.P836Q | missense SNV | NA | NA | 26.9 | D | D | D | 5.93 |
|  |  |  | 15 | 45409472 | *DUOXA2* | c.C738G:p.Y246X | nonsense | NA | 0.0025 | 34 | NA | NA | D | NA |
|  |  |  | 8 | 134146934 | *TG* | c.G8203A:p.G2735R | missense SNV | NA | 0 | 11.88 | T | D | N | NA |
| 197 | male | Athyreosis | 15 | 45409472 | *DUOXA2* | c.C738G:p.Y246X | nonsense | NA | 0.0025 | 34 | NA | NA | D | NA |
| 198 | male | Ectopia | 15 | 45391946 | *DUOX2* | c.G3329A:p.R1110Q | missense SNV | 0.002 | 0.0025 | 35 | D | D | D | 5.6 |
|  |  |  | 15 | 45412435 | *DUOXA1* | c.C503T:p.T168M | missense SNV | 0.002 | 0.0036 | 29.6 | D | D | D | 5.12 |
|  |  |  | 8 | 133913702 | *TG* | c.C3538T:p.Q1180X | nonsense | NA | NA | 37 | NA | NA | A | 5.12 |
| 199 | male | Athyreosis | 9 | 3828355 | *GLIS3* | c.G2710A:p.G904R | missense SNV | NA | 0 | 24.6 | D | D | D | 5.02 |
|  |  |  | 14 | 36987150 | *NKX2-1* | c.G449T:p.S150I | missense SNV | NA | NA | 24.2 | D | P | D | 3.72 |
|  |  |  | 8 | 133984055 | *TG* | c.C5992T:p.R1998X | nonsense | NA | 0 | 40 | NA | NA | A | 4.64 |
|  |  |  | 7 | 107355874 | *SLC26A4* | c.C2326T:p.R776C | missense SNV | NA | 0 | 34 | D | D | D | 5.76 |
| 200 | male | Athyreosis | 9 | 3828387 | *GLIS3* | c.C2678T:p.S893F | missense SNV | 0.003 | 0.0018 | 26.5 | D | D | D | 5.92 |
| 201 | male | Ectopia | 9 | 100617286 | *FOXE1* | c.G1090A:p.G364S | missense SNV | 0.001 | 0.0006 | 18.23 | T | D | D | 3.88 |
|  |  |  | 15 | 45387154 | *DUOX2* | c.G4375A:p.D1459N | missense SNV | 0.001 | 0.0006 | 34 | D | D | D | 5.41 |
| 202 | male | Gland-in-Situ | 9 | 3856011 | *GLIS3* | c.A2471G:p.H824R | missense SNV | 0.005 | 0.0071 | 23.1 | D | P | D | 5.8 |
|  |  |  | 15 | 45409472 | *DUOXA2* | c.C738G:p.Y246X | nonsense | NA | 0.0025 | 34 | NA | NA | D | NA |
| 203 | male | Gland-in-Situ | 15 | 45388079 | *DUOX2* | c.C4027T:p.L1343F | missense SNV | 0.005 | 0.0068 | 29.5 | T | P | D | 5.57 |
|  |  |  | 15 | 45398423 | *DUOX2* | c.G2048T:p.R683L | missense SNV | 0.003 | 0.0038 | 34 | D | D | D | 5.31 |
|  |  |  | 15 | 45400357 | *DUOX2* | c.G1462A:p.G488R | missense SNV | 0.001 | 0.0015 | 34 | D | D | D | 5.47 |
|  |  |  | 15 | 45411399 | *DUOXA1* | c.A802G:p.S268G | missense SNV | 0.0088 | 0.0032 | 23.4 | D | D | D | 5.31 |
| 204 | male | Ectopia | 15 | 45409472 | *DUOXA2* | c.C738G:p.Y246X | nonsense | NA | 0.0025 | 34 | NA | NA | D | NA |
| 205 | male | Gland-in-Situ | 15 | 45391946 | *DUOX2* | c.G3329A:p.R1110Q | missense SNV | 0.002 | 0.0025 | 35 | D | D | D | 5.6 |
|  |  |  | 15 | 45412435 | *DUOXA1* | c.C503T:p.T168M | missense SNV | 0.002 | 0.0036 | 29.6 | D | D | D | 5.12 |
| 206 | male | Ectopia | 7 | 107350577 | *SLC26A4* | c.A2168G:p.H723R | missense SNV | 0.002 | 0.0017 | 26.8 | D | D | A | 5.51 |
| 207 | male | Gland-in-Situ | 15 | 45390213 | *DUOX2* | c.G3559A:p.V1187I | missense SNV | NA | 0.0003 | 16.28 | T | B | D | 2.83 |
|  |  |  | 15 | 45436386 | *DUOX1* | c.C2089T:p.R697C | missense SNV | NA | 0.0001 | 24.2 | T | B | D | 4.78 |
|  |  |  | 7 | 107350577 | *SLC26A4* | c.A2168G:p.H723R | missense SNV | 0.002 | 0.0017 | 26.8 | D | D | A | 5.51 |
| 208 | male | Gland-in-Situ | 15 | 45388079 | *DUOX2* | c.C4027T:p.L1343F | missense SNV | 0.005 | 0.0068 | 29.5 | T | P | D | 5.57 |
|  |  |  | 15 | 45396158 | *DUOX2* | c.G2654T:p.R885L | missense SNV | 0.004 | 0.0044 | 27.5 | D | D | D | 4.29 |
|  |  |  | 15 | 45398423 | *DUOX2* | c.G2048T:p.R683L | missense SNV | 0.003 | 0.0038 | 34 | D | D | D | 5.31 |
|  |  |  | 15 | 45411399 | *DUOXA1* | c.A802G:p.S268G | missense SNV | 0.0088 | 0.0032 | 23.4 | D | D | D | 5.31 |
|  |  |  | 8 | 133935727 | *TG* | c.C4673T:p.A1558V | missense SNV | NA | NA | 11.27 | D | P | N | NA |
| 209 | male | Gland-in-Situ | 14 | 81609751 | *TSHR* | c.G1349A:p.R450H | missense SNV | 0.001 | 0.0034 | 32 | D | D | D | 5.74 |
|  |  |  | 14 | 81609958 | *TSHR* | c.G1556A:p.R519H | missense SNV | NA | 0.0001 | 33 | D | D | D | 5.46 |
| 210 | male | Hypoplasia | 3 | 24174797 | *THRB* | exon9:c.885+10T>C | splicing | NA | NA | 14.49 | NA | NA | NA | NA |
| 211 | male | Hypoplasia | 3 | 24174797 | *THRB* | exon9:c.885+10T>C | splicing | NA | NA | 14.49 | NA | NA | NA | NA |
| 212 | female | Ectopia | 15 | 45409472 | *DUOXA2* | c.C738G:p.Y246X | nonsense | NA | 0.0025 | 34 | NA | NA | D | NA |
| 213 | female | Athyreosis | 2 | 1497754 | *TPO* | c.G1949A:p.G650E | missense SNV | 0.001 | 0.0001 | 25.2 | D | D | D | 4.84 |
| 214 | male | Athyreosis | 15 | 45396158 | *DUOX2* | c.G2654T:p.R885L | missense SNV | 0.004 | 0.0044 | 27.5 | D | D | D | 4.29 |
| 215 | male | Athyreosis | 15 | 45409472 | *DUOXA2* | c.C738G:p.Y246X | nonsense | NA | 0.0025 | 34 | NA | NA | D | NA |
| 216 | female | Athyreosis | 15 | 45408316 | *DUOXA2* | exon3:c.206-6C>G | splicing | 0.002 | 0.0006 | 11.89 | NA | NA | NA | NA |
| 217 | female | Athyreosis | 14 | 81557414 | *TSHR* | c.G394C:p.G132R | missense SNV | NA | 0.0005 | 23.8 | T | D | D | 5.8 |
|  |  |  | 14 | 81558898 | *TSHR* | c.T491A:p.M164K | missense SNV | NA | NA | 27.9 | D | D | D | 5.28 |
| 218 | female | Athyreosis | 14 | 81609967 | *TSHR* | c.C1565T:p.A522V | missense SNV | NA | NA | 31 | D | D | D | 5.46 |
|  |  |  | 8 | 134128945 | *TG* | c.A7847T:p.N2616I | missense SNV | 0.0024 | 0.0016 | 12.35 | D | B | P | NA |
| 219 | female | Athyreosis | 15 | 45409472 | *DUOXA2* | c.C738G:p.Y246X | nonsense | NA | 0.0025 | 34 | NA | NA | D | NA |

NA: not available; SNV: single nucleotide variant; SIFT：D- Deleterious, T- Tolerated; Polyphen2: D- Probably damaging, P- Possibly damaging, B- Benign; Mutation Taster: A- Disease causing automatic, D- Disease causing, N– Polymorphism, P– Polymorphism automatic.
