# Supplementary material for: DUOX2 and DUOXA2 Variants Confer Susceptibility to Thyroid Dysgenesis and Gland-in-situ With Congenital Hypothyroidism
Source: Front Endocrinol (Lausanne). 2020 Apr 21;11:237. doi: 10.3389/fendo.2020.00237 (PMC7212429; doi:10.3389/fendo.2020.00237)
Supplement: Supplementary file 2 [file Table_2.DOCX]

Table S2: Basic information of 168 identified mutation sites.

| Chromosome | Position | Gene | Varient | Varient Type | Novel or Reported | rs numbers | AF in Chinese population |
| --- | --- | --- | --- | --- | --- | --- | --- |
| 2 | 1426892 | *TPO* | c.C170T:p.T57M | missense SNV | Novel |  | NA |
| 2 | 1457597 | *TPO* | exon5:c.612+2T>G | splicing | Novel |  | NA |
| 2 | 1480873 | *TPO* | c.C835T:p.R279W | missense SNV | Reported | rs762810333 | NA |
| 2 | 1481101 | *TPO* | c.C1063T:p.R355C | missense SNV | Novel |  | NA |
| 2 | 1481120 | *TPO* | c.G1082T:p.R361L | missense SNV | Reported | rs201781919 | 0.023256 |
| 2 | 1497754 | *TPO* | c.G1949A:p.G650E | missense SNV | Reported | rs200122184 | 0.001661 |
| 2 | 1499831 | *TPO* | c.C1906T:p.R636W | missense SNV | Reported | rs121908087 | NA |
| 2 | 1500456 | *TPO* | c.C2134T:p.R712W | missense SNV | Reported | rs114406277 | 0.004983 |
| 2 | 1520672 | *TPO* | c.C2365T:p.R789W | missense SNV | Reported | rs28913014 | 0.003322 |
| 2 | 1520714 | *TPO* | c.G2407A:p.G803R | missense SNV | Reported | rs556552435 | 0 |
| 2 | 1544412 | *TPO* | c.G2494A:p.G832R | missense SNV | Reported | rs546683738 | 0 |
| 9 | 3828312 | *GLIS3* | c.G2753A:p.R918H | missense SNV | Reported | rs147357710 | 0 |
| 9 | 3828319 | *GLIS3* | c.G2746A:p.V916M | missense SNV | Reported | rs151140581 | 0 |
| 9 | 3828355 | *GLIS3* | c.G2710A:p.G904R | missense SNV | Reported | rs150310830 | 0 |
| 9 | 3828387 | *GLIS3* | c.C2678T:p.S893F | missense SNV | Reported | rs201132217 | 0.004983 |
| 9 | 3829459 | *GLIS3* | c.C2507A:p.P836Q | missense SNV | Novel |  | NA |
| 9 | 3829462 | *GLIS3* | c.C2504T:p.P835L | missense SNV | Reported | rs766517772 | NA |
| 9 | 3856011 | *GLIS3* | c.A2471G:p.H824R | missense SNV | Reported | rs140981222 | 0 |
| 9 | 3879532 | *GLIS3* | c.C2192G:p.P731R | missense SNV | Novel |  | NA |
| 9 | 4118540 | *GLIS3* | c.G938C:p.G313A | missense SNV | Reported | rs35154632 | 0.003322 |
| 9 | 4286137 | *GLIS3* | c.C289T:p.R97X | nonsense | Reported | rs780019691 | NA |
| 19 | 17986789 | *SLC5A5* | c.A572G:p.D191G | missense SNV | Novel |  | NA |
| 19 | 17992867 | *SLC5A5* | c.T1157G:p.I386S | missense SNV | Novel |  | NA |
| 19 | 17992872 | *SLC5A5* | c.A1162T:p.K388X | nonsense | Novel |  | NA |
| 3 | 24174797 | *THRB* | exon9:c.885+10T>C | splicing | Novel |  | NA |
| 3 | 24185051 | *THRB* | c.G679A:p.E227K | missense SNV | Novel |  | NA |
| 3 | 24231741 | *THRB* | c.G107A:p.C36Y | missense SNV | Reported | rs758677446 | NA |
| 3 | 24231772 | *THRB* | c.G76A:p.D26N | missense SNV | Reported | rs776036533 | NA |
| 14 | 36986583 | *NKX2-1* | c.C1016T:p.A339V | missense SNV | Reported | rs537209983 | 0.003322 |
| 14 | 36986635 | *NKX2-1* | c.G964A:p.G322S | missense SNV | Reported | rs200560568 | 0.003322 |
| 14 | 36987150 | *NKX2-1* | c.G449T:p.S150I | missense SNV | Novel |  | NA |
| 14 | 36988426 | *NKX2-1* | c.C137T:p.A46V | missense SNV | Novel |  | NA |
| 15 | 45386428 | *DUOX2* | c.A4567G:p.T1523A | missense SNV | Reported | rs149306918 | 0.001661 |
| 15 | 45386458 | *DUOX2* | c.G4537C:p.G1513R | missense SNV | Novel |  | NA |
| 15 | 45386877 | *DUOX2* | c.C4408T:p.R1470W | missense SNV | Reported | rs200785525 | 0.006645 |
| 15 | 45386880 | *DUOX2* | c.G4405A:p.E1469K | missense SNV | Reported | rs376623263 | 0 |
| 15 | 45387154 | *DUOX2* | c.G4375A:p.D1459N | missense SNV | Reported | rs199546504 | 0.001661 |
| 15 | 45387181 | *DUOX2* | c.T4348C:p.Y1450H | missense SNV | Reported | rs753591292 | NA |
| 15 | 45387642 | *DUOX2* | c.G4232A:p.C1411Y | missense SNV | Reported | rs766067391 | 45387642 |
| 15 | 45387781 | *DUOX2* | c.G4093A:p.G1365R | missense SNV | Novel |  | NA |
| 15 | 45388060 | *DUOX2* | c.C4046G:p.S1349C | missense SNV | Reported | rs768030115 | NA |
| 15 | 45388079 | *DUOX2* | c.C4027T:p.L1343F | missense SNV | Reported | rs147945181 | 0.004983 |
| 15 | 45388243 | *DUOX2* | c.A3863C:p.Q1288P | missense SNV | Novel |  | NA |
| 15 | 45389484 | *DUOX2* | c.C3799T:p.R1267W | missense SNV | Reported | rs752437461 | NA |
| 15 | 45389889 | *DUOX2* | c.G3616A:p.A1206T | missense SNV | Reported | rs762588205 | NA |
| 15 | 45390213 | *DUOX2* | c.G3559A:p.V1187I | missense SNV | Reported | rs138971379 | 0 |
| 15 | 45391884 | *DUOX2* | c.G3391T:p.A1131S | missense SNV | Reported | rs147540920 | NA |
| 15 | 45391946 | *DUOX2* | c.G3329A:p.R1110Q | missense SNV | Reported | rs368488511 | 0.001661 |
| 15 | 45392024 | *DUOX2* | c.G3251A:p.R1084Q | missense SNV | Reported | rs558919433 | 0 |
| 15 | 45393403 | *DUOX2* | c.G2921A:p.R974H | missense SNV | Reported | rs778216481 | NA |
| 15 | 45393424 | *DUOX2* | c.T2900C:p.I967T | missense SNV | Reported | rs765025859 | NA |
| 15 | 45394177 | *DUOX2* | c.G2665A:p.E889K | missense SNV | Novel |  | NA |
| 15 | 45396158 | *DUOX2* | c.G2654T:p.R885L | missense SNV | Reported | rs181461079 | 0.006645 |
| 15 | 45396177 | *DUOX2* | c.G2635A:p.E879K | missense SNV | Reported | rs774556391 | NA |
| 15 | 45396517 | *DUOX2* | c.C2381T:p.S794F | missense SNV | Novel |  | NA |
| 15 | 45396563 | *DUOX2* | c.G2335A:p.V779M | missense SNV | Reported | rs145061993 | 0.003322 |
| 15 | 45396571 | *DUOX2* | exon19:c.2335-8G>A | splicing | Reported | rs532139907 | 0.001661 |
| 15 | 45397861 | *DUOX2* | c.T2314C:p.F772L | missense SNV | Novel |  | NA |
| 15 | 45397884 | *DUOX2* | c.G2291A:p.R764Q | missense SNV | Reported | rs201884203 | 0 |
| 15 | 45397885 | *DUOX2* | c.C2290T:p.R764W | missense SNV | Reported | rs141291775 | 0 |
| 15 | 45397973 | *DUOX2* | c.G2202A:p.W734X | nonsense | Reported | rs769789467 | NA |
| 15 | 45398370 | *DUOX2* | c.C2101T:p.R701X | nonsense | Reported | rs201109959 | NA |
| 15 | 45398423 | *DUOX2* | c.G2048T:p.R683L | missense SNV | Reported | rs8028305 | 0.001661 |
| 15 | 45398798 | *DUOX2* | c.C1873T:p.R625X | nonsense | Reported | rs770083296 | NA |
| 15 | 45399153 | *DUOX2* | c.C1708T:p.Q570X | nonsense | Novel |  | NA |
| 15 | 45400357 | *DUOX2* | c.G1462A:p.G488R | missense SNV | Reported | rs191759494 | 0 |
| 15 | 45401075 | *DUOX2* | c.G1310C:p.G437A | missense SNV | Reported | rs769796932 | NA |
| 15 | 45401724 | *DUOX2* | c.G1232A:p.R411K | missense SNV | Reported | rs764353021 | NA |
| 15 | 45402140 | *DUOX2* | c.G1079T:p.G360V | missense SNV | Reported | rs762148198 | NA |
| 15 | 45402159 | *DUOX2* | c.C1060T:p.R354W | missense SNV | Reported | rs766496010 | NA |
| 15 | 45402165 | *DUOX2* | c.C1054T:p.H352Y | missense SNV | Novel |  | NA |
| 15 | 45403623 | *DUOX2* | c.C674T:p.P225L | missense SNV | Novel |  | NA |
| 15 | 45403784 | *DUOX2* | exon6:c.514-1G>A | splicing | Novel |  | NA |
| 15 | 45403787 | *DUOX2* | exon6:c.514-4G>A | splicing | Reported | rs374243549 | NA |
| 15 | 45404082 | *DUOX2* | c.A397T:p.I133F | missense SNV | Novel |  | NA |
| 15 | 45404115 | *DUOX2* | c.C364A:p.P122T | missense SNV | Reported | rs200265605 | 0.001661 |
| 15 | 45404850 | *DUOX2* | c.C227T:p.P76L | missense SNV | Reported | rs767705906 | NA |
| 15 | 45406818 | *DUOXA2* | c.C15A:p.N5K | missense SNV | Reported | rs574384446 | NA |
| 15 | 45408059 | *DUOXA2* | exon2:c.205+5G>A | splicing | Novel |  | NA |
| 15 | 45408316 | *DUOXA2* | exon3:c.206-6C>G | splicing | Reported | rs190250575 | 0.001661 |
| 15 | 45408396 | *DUOXA2* | c.C280T:p.R94C | missense SNV | Novel |  | NA |
| 15 | 45408819 | *DUOXA2* | c.G446A:p.G149E | missense SNV | Reported | rs762481523 | NA |
| 15 | 45409307 | *DUOXA2* | c.G573A:p.W191X | nonsense | Novel |  | NA |
| 15 | 45409472 | *DUOXA2* | c.C738G:p.Y246X | nonsense | Reported | rs4774518 | NA |
| 15 | 45411399 | *DUOXA1* | c.A802G:p.S268G | missense SNV | Reported | rs16977686 | 0.018272 |
| 15 | 45412435 | *DUOXA1* | c.C503T:p.T168M | missense SNV | Reported | rs149960164 | 0.003322 |
| 15 | 45414422 | *DUOXA1* | c.C166T:p.R56W | missense SNV | Reported | rs117062041 | 0.004983 |
| 15 | 45436386 | *DUOX1* | c.C2089T:p.R697C | missense SNV | Reported | rs765746160 | NA |
| 15 | 45440145 | *DUOX1* | c.C2592A:p.D864E | missense SNV | Novel |  | NA |
| 15 | 45442895 | *DUOX1* | c.A2884T:p.I962F | missense SNV | Reported | rs755801190 | NA |
| 15 | 45444175 | *DUOX1* | c.A3118C:p.N1040H | missense SNV | Novel |  | NA |
| 15 | 45444526 | *DUOX1* | c.C3236A:p.T1079N | missense SNV | Novel |  | NA |
| 15 | 45454115 | *DUOX1* | c.C4036G:p.L1346V | missense SNV | Novel |  | NA |
| 15 | 45457004 | *DUOX1* | c.G4561A:p.G1521S | missense SNV | Novel |  | NA |
| 14 | 81554378 | *TSHR* | exon4:c.392+6T>G | splicing | Novel |  | NA |
| 14 | 81557414 | *TSHR* | c.G394C:p.G132R | missense SNV | Reported | rs760874290 | NA |
| 14 | 81557483 | *TSHR* | c.A463G:p.I155V | missense SNV | Reported | rs141293178 | NA |
| 14 | 81558873 | *TSHR* | exon6:c.468-2A>G | splicing | Reported | rs779806385 | NA |
| 14 | 81558898 | *TSHR* | c.T491A:p.M164K | missense SNV | Novel |  | NA |
| 14 | 81563048 | *TSHR* | c.C611T:p.A204V | missense SNV | Reported | rs760702366 | NA |
| 14 | 81606153 | *TSHR* | c.G823A:p.A275T | missense SNV | Reported | rs180762551 | 0.001661 |
| 14 | 81609317 | *TSHR* | c.T915A:p.S305R | missense SNV | Reported | rs142122217 | 0.004983 |
| 14 | 81609426 | *TSHR* | c.A1024T:p.K342X | nonsense | Novel |  | NA |
| 14 | 81609571 | *TSHR* | c.G1169T:p.C390F | missense SNV | Reported | rs371139156 | NA |
| 14 | 81609640 | *TSHR* | c.G1238A:p.G413D | missense SNV | Novel |  | NA |
| 14 | 81609672 | *TSHR* | c.G1270T:p.V424F | missense SNV | Reported | rs587778742 | NA |
| 14 | 81609750 | *TSHR* | c.C1348T:p.R450C | missense SNV | Novel |  | NA |
| 14 | 81609751 | *TSHR* | c.G1349A:p.R450H | missense SNV | Reported | rs189261858 | 0.001661 |
| 14 | 81609775 | *TSHR* | c.T1373A:p.F458Y | missense SNV | Novel |  | NA |
| 14 | 81609958 | *TSHR* | c.G1556A:p.R519H | missense SNV | Reported | rs780018604 | NA |
| 14 | 81609967 | *TSHR* | c.C1565T:p.A522V | missense SNV | Novel |  | NA |
| 14 | 81609976 | *TSHR* | c.T1574C:p.F525S | missense SNV | Reported | rs200138601 | 0.001661 |
| 14 | 81609984 | *TSHR* | c.C1582A:p.R528S | missense SNV | Novel |  | NA |
| 14 | 81610468 | *TSHR* | c.T2066G:p.V689G | missense SNV | Reported | rs761341933 | NA |
| 14 | 81610674 | *TSHR* | c.G2272A:p.E758K | missense SNV | Reported | rs746522401 | NA |
| 9 | 100616530 | *FOXE1* | c.C334T:p.L112F | missense SNV | Reported | rs757519887 | NA |
| 9 | 100616789 | *FOXE1* | c.C593G:p.A198G | missense SNV | Novel |  | NA |
| 9 | 100616878 | *FOXE1* | c.C682G:p.P228A | missense SNV | Reported | rs556903865 | 0 |
| 9 | 100616953 | *FOXE1* | c.G757A:p.G253S | missense SNV | Reported | rs778490079 | NA |
| 9 | 100617170 | *FOXE1* | c.A974C:p.Y325S | missense SNV | Reported | rs779912895 | NA |
| 9 | 100617286 | *FOXE1* | c.G1090A:p.G364S | missense SNV | Reported | rs565079882 | 0.001661 |
| 7 | 107302185 | *SLC26A4* | c.G99T:p.Q33H | missense SNV | Novel |  | NA |
| 7 | 107314782 | *SLC26A4* | c.G589A:p.G197R | missense SNV | Reported | rs111033380 | NA |
| 7 | 107323898 | *SLC26A4* | exon8:c.919-2A>G | splicing | Reported | rs111033313 | NA |
| 7 | 107330644 | *SLC26A4* | c.C1225T:p.R409C | missense SNV | Reported | rs147952620 | 0 |
| 7 | 107330645 | *SLC26A4* | c.G1226A:p.R409H | missense SNV | Reported | rs111033305 | NA |
| 7 | 107342443 | *SLC26A4* | c.G1975C:p.V659L | missense SNV | Reported | rs200455203 | 0 |
| 7 | 107342497 | *SLC26A4* | c.C2029T:p.R677W | missense SNV | Reported | rs397516426 | NA |
| 7 | 107350577 | *SLC26A4* | c.A2168G:p.H723R | missense SNV | Reported | rs121908362 | 0.001661 |
| 7 | 107355874 | *SLC26A4* | c.C2326T:p.R776C | missense SNV | Reported | rs111033255 | 0 |
| 2 | 113984778 | *PAX8* | c.C1064T:p.A355V | missense SNV | Reported | rs145036350 | 0 |
| 2 | 113994290 | *PAX8* | c.C786A:p.Y262X | nonsense | Novel |  | NA |
| 2 | 113999158 | *PAX8* | c.T747G:p.Y249X | nonsense | Novel |  | NA |
| 2 | 114002108 | *PAX8* | c.C285G:p.Y95X | nonsense | Novel |  | NA |
| 8 | 133880359 | *TG* | exon2:c.68-1G>A | splicing | Novel |  | NA |
| 8 | 133894843 | *TG* | c.C875T:p.S292F | missense SNV | Novel |  | NA |
| 8 | 133898968 | *TG* | c.C1351T:p.R451X | nonsense | Reported | rs773142559 | NA |
| 8 | 133899061 | *TG* | c.C1444T:p.Q482X | nonsense | Novel |  | NA |
| 8 | 133900333 | *TG* | c.C2281T:p.P761S | missense SNV | Reported | rs184309049 | 0 |
| 8 | 133900411 | *TG* | c.C2359T:p.R787X | nonsense | Reported | rs752966476 | NA |
| 8 | 133909932 | *TG* | c.G3040C:p.D1014H | missense SNV | Reported | rs114772213 | 0.001661 |
| 8 | 133910424 | *TG* | c.G3150A:p.W1050X | nonsense | Novel |  | NA |
| 8 | 133910471 | *TG* | c.G3197A:p.R1066H | missense SNV | Reported | rs116119508 | 0 |
| 8 | 133913702 | *TG* | c.C3538T:p.Q1180X | nonsense | Novel |  | NA |
| 8 | 133919106 | *TG* | c.C3808T:p.R1270C | missense SNV | Reported | rs754517497 | NA |
| 8 | 133919133 | *TG* | c.C3835T:p.R1279W | missense SNV | Reported | rs368926894 | 0 |
| 8 | 133923622 | *TG* | c.G4003A:p.V1335M | missense SNV | Novel |  | NA |
| 8 | 133923770 | *TG* | c.A4151C:p.H1384P | missense SNV | Novel |  | NA |
| 8 | 133925442 | *TG* | c.G4310A:p.W1437X | nonsense | Novel |  | NA |
| 8 | 133935727 | *TG* | c.C4673T:p.A1558V | missense SNV | Novel |  | NA |
| 8 | 133948050 | *TG* | c.G4982A:p.R1661H | missense SNV | Reported | rs115509019 | 0 |
| 8 | 133980165 | *TG* | c.G5813T:p.G1938V | missense SNV | Novel |  | NA |
| 8 | 133981767 | *TG* | c.A5928G:p.I1976M | missense SNV | Reported | rs374368216 | NA |
| 8 | 133984042 | *TG* | c.C5979A:p.F1993L | missense SNV | Novel |  | NA |
| 8 | 133984055 | *TG* | c.C5992T:p.R1998X | nonsense | Reported | rs777075341 | NA |
| 8 | 134024144 | *TG* | exon36:c.6263-2A>G | splicing | Novel |  | NA |
| 8 | 134030184 | *TG* | c.G6401A:p.C2134Y | missense SNV | Reported | rs763309772 | NA |
| 8 | 134107412 | *TG* | c.G7364A:p.R2455H | missense SNV | Reported | rs2272707 | 0 |
| 8 | 134107420 | *TG* | c.C7372T:p.P2458S | missense SNV | Reported | rs201280992 | 0.001661 |
| 8 | 134125826 | *TG* | c.G7733A:p.R2578Q | missense SNV | Reported | rs115015716 | 0.001661 |
| 8 | 134125846 | *TG* | c.C7753T:p.R2585W | missense SNV | Reported | rs114211101 | 0.006645 |
| 8 | 134128945 | *TG* | c.A7847T:p.N2616I | missense SNV | Reported | rs10091530 | 0.006645 |
| 8 | 134146934 | *TG* | c.G8203A:p.G2735R | missense SNV | Reported | rs777891366 | NA |
| 6 | 150713627 | *IYD* | c.C517A:p.L173I (NM_001164695;NM_203395) | missense SNV | Novel |  | NA |
| 6 | 150716549 | *IYD* | c.T707C:p.L236P (NM_001164695) | missense SNV | Reported | rs574170213 | 0.001661 |
| 6 | 150719321 | *IYD* | c.C818T:p.T273M (NM_203395) | missense SNV | Reported | rs141250255 | 0 |
| 5 | 172659675 | *NKX2-5* | c.A872T:p.N291I | missense SNV | Novel |  | NA |
| 5 | 172659699 | *NKX2-5* | c.C848A:p.P283Q | missense SNV | Reported | rs375086983 | NA |
